# Phytochemical Characterization of *Euphorbia peplis* Extracts and Their Antioxidant, Enzyme Inhibitory, and Cytotoxic Activities: An Integrated Experimental and In Silico Study

**DOI:** 10.1002/fsn3.72244

**Published:** 2026-08-26

**Authors:** Sakina Yagi, Esraa A. Elhawary, Omayma A. Eldahshna, Abdel Nasser B. Singab, Mehmet Veysi Cetiz, Abdullahi Ibrahim Uba, Ismail Yapıcı, Ilhami Gulcin, Eliana Fernandes, Luísa Custódio, Maria João Rodrigues, Evren Yildiztugay, Gokhan Zengin

**Affiliations:** ^1^ Department of Botany, Faculty of Science University of Khartoum Khartoum Sudan; ^2^ Pharmacognosy Department, Faculty of Pharmacy Ain Shams University Cairo Egypt; ^3^ Department of Medical Biochemistry, Faculty of Medicine Harran University Sanliurfa Turkey; ^4^ Department of Biostatistics and Medical Informatics, Faculty of Medicine İstinye University Istanbul Turkey; ^5^ Department of Chemistry, Science Faculty Ataturk University Erzurum Turkey; ^6^ Centro de Ciências do Mar do Algarve (CCMAR/CIMAR LA), Campus de Gambelas Universidade do Algarve Faro Portugal; ^7^ Department of Biotechnology, Science Faculty Selcuk University Konya Turkey; ^8^ Department of Biology, Science Faculty Selcuk University Konya Turkey

**Keywords:** antioxidant, Cytotoxicity, enzyme inhibition, *Euphorbia peplis*, multivariate data analysis, phytoconstituents

## Abstract

Euphorbia members (Euphorbiaceae) are valuable sources of lead compounds for potential drug discovery. This study was conducted to evaluate, for the first time, the phytoconstituents, antioxidant capacity, enzyme inhibitory, and cytotoxic properties of *E. peplis* . Extracts were prepared from the aerial parts using ethyl acetate (EtOAc), ethanol (EtOH), 70% EtOH, and water. Results showed that the 70% EtOH and EtOH extracts contained the highest levels of total phenolics (68.12 mg GAE/g) and flavonoids (45.49 mg RE/g). UPLC‐ESI‐MSn analysis revealed a variety of phytochemicals, including flavonoids, cinnamic acid derivatives, tannins, triterpenoids, and saponins, with 24 metabolites tentatively identified. PCA grouped these metabolites into three clusters, and their distribution was visualized with a heatmap. Polar extracts demonstrated strong antioxidant activity, with the 70% EtOH extract showing the highest values in most assays (DPPH = 385.50 mg TE/g; ABTS = 466.31 mg TE/g; CUPRAC = 439.95 mg TE/g; FRAP = 305.07 mg TE/g; PBD = 2.45 mmol TE/g). The EtOH and EtOAc extracts exhibited the strongest anti‐acetylcholinesterase (2.83 mg GALAE/mg) and anti‐butyrylcholinesterase (2.18 mg GALAE/mg) activities, respectively. Both the 70% EtOH and EtOH extracts showed the best anti‐tyrosinase effects (54.40 and 53.39 mg KAE/g; *p* ≥ 0.05). The EtOAc extract was more toxic toward SHSY5Y cells, with a viability of 4.31%, compared to 8.18% in normal KEK293 cells. Network pharmacology identified 11 common targets for *E. peplis* metabolites, with AKT1, EGFR, GSK3B, ESR1/ESR2, and CCND1 serving as key hubs. Pathway enrichment analysis highlighted PI3K‐Akt, EGFR, and hormone‐related pathways. Docking and molecular dynamics simulations confirmed stable multi‐target binding. These findings suggest that *E. peplis* could be a promising source of antioxidants and compounds with potential anticancer and enzyme‐inhibitory activities relevant to human diseases.

## Graphical abstract

*Euphorbia peplis* extracts may act as versatile components in the development of health‐promoting applications.

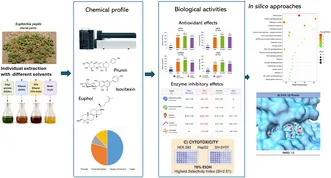

## 1 Introduction

Bioactive substances with a variety of biological activities, including antioxidant, anticancer, antidiabetic, and anti‐inflammatory properties, can be found in natural products (Aly et al. 2022; El‐Nashar et al. 2022; Mostafa et al. 2016). They are crucial leads for drug discovery and the creation of safer, more potent therapeutic agents because of their structural diversity and natural origin.

The genus *Euphorbia* is one of the largest genera of flowering plants within the spurge family (Euphorbiaceae), containing approximately 2000 species, of which around 800 are succulent plants (Walker 2023). It is mainly distributed in tropical and subtropical areas of the world. *Euphorbia* species are characterized by the production of a milky latex and specialized inflorescences (cyathia) (Benjamaa et al. 2022). They are widely used in traditional medicine to treat a range of ailments, including respiratory infections, body and skin irritations, digestive complaints, inflammatory infections, body pain, microbial illness, snake or scorpion bites, among others (Kemboi et al. 2020). They have diverse secondary metabolites, and about 455 diterpenoids were isolated from 35 *Euphorbia* species, including polycyclic and macrocyclic diterpenes like jatrophane, ingenane, daphnane, tigliane, and lathyrane (Vasas and Hohmann 2014; Xu et al. 2021). Additionally, 130 different types of triterpenes have been isolated, mainly tetracyclic triterpenoids like tirucallane, euphane, lanostane, and cycloartanes. Also, pentacyclic triterpenoids such as lupane, oleanane, taraxarane, friedoursane, friedelane, and ursane triterpenoids have been reported in genus (Xu et al. 2021). A detailed review article showed that *Euphorbia* species possess diverse biological activities like antibacterial, antifungal, antiviral, anti‐inflammatory, anticancer, antimalarial, anti‐Alzheimer, and anthelmintic activities (Xu et al. 2021).

Despite this chemical richness, most studies have focused on a limited number of *Euphorbia* species (e.g., *E. hirta, E. peplus, E. tirucalli*), while many taxa remain chemically and biologically underexplored. Among them, *E. peplis* is an annual, prostrate herb that grows in sandy and gravelly coastal habitats and is often confused with *E. peplus* , although both species differ clearly in morphology and growth habit. *E. peplis* is characterized by a circular arrangement of four to six reddish stems, glaucous heart‐shaped leaves, and solitary flowers located at leaf axils, producing globular capsule fruits (Mali and Panchal 2017; Pahlevani et al. 2015). Ethnomedicinally, members of the genus have been widely used across Europe, Africa, and Asia as purgatives, emetics, diuretics, and topical treatments for skin diseases, tumors, and inflammatory conditions (Vasas and Hohmann 2014; Sulaiman et al. 2020). However, in contrast to well‐studied congeners such as *E. hirta* and *E. peplus* , the phytochemical composition and pharmacological profile of *E. peplis* remain largely unexplored, with only fragmentary reports.

Previous phytochemical investigations on *Euphorbia* species have revealed abundant terpenoids, flavonoids, sterols, and phenolic acids. LC–MS‐based profiling of species such as *E. hirta, E. peplus, E* *. antiquorum* , and *E. tirucalli* has led to the identification of numerous ingenane‐ and jatrophane‐type diterpenoids, flavonoid glycosides, and triterpenoid derivatives (Xie et al. 2021, 2020). For *E. peplis* , preliminary screening has only indicated the presence of limited compounds such as quercetin and kaempferol glycosides, caffeic and ferulic acids, and triterpenoids including lupeol and β‐sitosterol (Elshamy et al. 2019). However, no comprehensive multi‐assay evaluation integrating phytochemical profiling with antioxidant, enzyme inhibitory, cytotoxic, and multivariate statistical analyses has been reported for this species.

To the best of our knowledge, this study represents the first integrated and comparative investigation of *E. peplis* aerial parts combining (i) detailed phytochemical characterization, (ii) multivariate data analysis to correlate chemical composition with bioactivity, and (iii) a side‐by‐side evaluation of antioxidant, enzyme inhibitory, and cytotoxic properties across multiple extracts. While previous studies on other *Euphorbia* species have mainly focused on isolated extracts or single bioactivity endpoints, the novelty of the present work lies in its systematic multi‐target and multi‐extract approach, allowing the identification of extract‐specific bioactivity patterns and potential structure–activity relationships within this underexplored species.

Antioxidant activity was assessed through complementary mechanisms, including free radical scavenging, metal chelation, and reducing power assays. Enzyme inhibitory potential was evaluated against key therapeutic targets, namely acetylcholinesterase (AChE), butyrylcholinesterase (BChE), tyrosinase, α‐amylase, α‐glucosidase, and carbonic anhydrase isoenzymes I and II. In addition, cytotoxic effects were examined against human embryonic kidney (HEK) 293 cells, hepatocellular carcinoma (HepG2) cells, and human neuroblastoma SH‐SY5Y cells. Furthermore, given that the molecular mechanisms underlying the bioactivity of *E. peplis* remain unknown, this study provides a basis for future network pharmacology, molecular docking, and molecular dynamics simulations to elucidate potential multi‐target interactions of its phytochemical constituents. Overall, this work fills a clear gap in the current literature by providing the first comprehensive chemical–biological correlation study of *E. peplis* , thereby establishing its potential as a previously overlooked source of multifunctional bioactive compounds.

## 2 Materials and Methods

### 2.1 Plant Collection

Plant specimens were collected in 2022 from the Lara beach area (Antalya, Turkey) at an elevation of 3 m. Dr. Evren Yildiztugay carried out the formal botanical classification of the material. A voucher specimen, under the code EY‐3207, was placed in the Faculty of Science at Selçuk University. After collection, the aerial parts were immediately separated and air‐dried at room temperature (25°C ± 2°C) in a shaded area (away from direct sunlight) and in a well‐ventilated area for about 10 days. After complete drying, the plant material was milled to a fine powder. To maintain chemical stability and prevent degradation, the powdered samples were placed in opaque containers, sealed, and stored under controlled conditions in the dark at approximately 25°C.

### 2.2 Plant Material Extraction

Four solvent systems were used to obtain bioactive constituents: ethyl acetate (EtOAc), ethanol (EtOH), 70% (v/v) ethanol–water, and distilled water (Aq). For each extraction, 10 g of the prepared plant material was mixed with 200 mL of the corresponding solvent. The methodology was adapted according to the properties of the solvent. Extractions using organic solvents were performed via maceration for 24 h under ambient conditions. In contrast, the water‐based extraction utilized a 15‐min infusion with heated water. Following extraction, the products obtained were concentrated using distinct techniques. The aqueous extract was stabilized by lyophilisation, while the organic solvent fractions were recovered by evaporating the solvents under reduced pressure using a rotary evaporator (Yagi et al. 2026).

### 2.3 Determination of Total Phenolic and Flavonoid Content

Phenolic compounds have significant biological activities, from antioxidant effects to anticancer properties. Thus, their level can provide the first insight into the potential uses of plant extracts. In this sense, total phenolic and flavonoid contents of the extracts were determined using established colorimetric assays, following the referenced procedure. Calibration curves were constructed using gallic acid (mg gallic acid equivalents (GAE)/g) and rutin (mg rutin equivalents (RE)/g) as standards (Bibi Sadeer et al. 2020). All experimental details are given in the supplemental materials.

### 2.4 UPLC/MS^n^ Analysis

The phytochemical analysis of the extracts was assessed according to the previously reported method using high‐performance liquid chromatographic (HPLC) analysis joined with an ESI‐MS/MS spectrometer detector (Yagi et al. 2024). This technique allowed tentative identification of phytoconstituents based on the molecular weights. The dried extract was reconstituted in HPLC‐grade methanol to obtain a final concentration of 100 μg/mL was then filtered via a membrane disc (0.20 μm). Then, the filtrate (10 μL) was injected into HPLC‐ESI‐MS/MS. The used HPLC instrument has the following specifications: Waters stocked with a reversed‐phase C‐18 column (ACQUITY UPLC‐BEH C‐18, particle size ~1.7 μm, dimensions = 2.1 × 50 mm). Before injection, the mobile phase was filtered through a membrane disc filter (0.2 μm) and sonicated. The elution run took 35 min using gradient elution (water (40%) and methanol (60%) acidified with 0.1% formic acid) with a flow rate of 0.2 mL/min. On an XEVO TQD triple quadrupole instrument, positive and negative ions were acquired using ESI‐MS. Waters Corporation, Milford, MA 01757, U.S.A. supplied the HPLC unit and mass spectrometer. Edwards, U.S.A., provided the vacuum pump at desolvation temperatures of 150°C and 440°C. The mass spectra were obtained using the software Masslynx 4.1 at an ESI range m/z of 100–1000. To tentatively identify the obtained mass spectra, the peak retention time (*R*_*t*_) and their fragmentation pattern were compared with the reported data in the literature. Metabolite annotation was considered putative and was based on low‐resolution LC–MS/MS data, including precursor ion masses, characteristic fragmentation patterns, and comparison with published literature and databases. No authentic standards or high‐resolution mass spectrometric measurements were used for confirmation; therefore, compound assignments should be regarded as tentative annotations.

### 2.5 Multivariate Data Analysis

The unsupervised principal component analysis (PCA) was performed using Unscrambler X 10.3 (CAMO SA, Oslo, Norway). A clustered heat map was built using NCSS. 12 software with Euclidean distance and the unweighted pair group method (Elhawary et al. 2021, 2025). PCA and clustered heat map analyses were performed using a binary matrix in which detected metabolites were coded as present (1) or absent (0) in each extract. The purpose of these analyses was to compare metabolite distribution patterns among extracts rather than to assess quantitative differences in metabolite abundance.

### 2.6 Biological Assays

#### 2.6.1 Antioxidant Properties

The term “antioxidants” encompasses multiple mechanisms, so no single universal method exists. In this sense, antioxidant capacities of the tested extracts were assessed using a panel of in vitro assays, following a previously described protocol (Grochowski et al. 2017). Reducing power was evaluated using FRAP (ferric reducing antioxidant power) and CUPRAC (cupric reducing antioxidant capacity), while radical scavenging was measured using DPPH (2,2‐diphenyl‐1‐picrylhydrazyl) and ABTS (2,2′‐azino‐bis(3‐ethylbenzothiazoline‐6‐sulfonic acid)) assays. Results from these four assays were quantified and standardized to Trolox and reported as mg Trolox equivalents (TE) per g of dried extract (mg TE/g). Trolox and expressed as milligrams of Trolox equivalent per gram of dried extract (mg TE/g). Total antioxidant capacity was additionally determined by the phosphomolybdenum method (PBD assay) and expressed as mmol Trolox equivalents per g (mmol TE/g). Finally, metal‐chelating activity was evaluated using a chelation assay and reported as mg EDTA equivalents (EDTAE) per g of extract (mg EDTAE/g). All experimental details are given in the supplemental materials.

#### 2.6.2 Enzyme Inhibitory Effects

The enzyme inhibition theory links to managing global health problems including Alzheimer's disease, diabetes and skin disorders. In this context, the enzyme inhibitory potential of the tested extracts was evaluated against five key targets: acetylcholinesterase (AChE), butyrylcholinesterase (BChE), tyrosinase, α‐amylase and α‐glucosidase using established colorimetric procedures (Grochowski et al. 2017). To standardize the results, inhibition was quantified using established reference compounds. Cholinesterase inhibitory activity was expressed as mg galanthamine equivalents (GALAE) per g of extract (mg GALAE/g). α‐Amylase and α‐glucosidase inhibitory activities were expressed as mg acarbose equivalents (ACAE) per g (mg ACAE/g), while tyrosinase inhibition was reported as mg kojic acid equivalents (KAE) per g (mg KAE/g). In addition, the extracts were evaluated for inhibitory activity against human carbonic anhydrase isoenzymes I and II (hCA I and hCA II). All experimental details are given in the supplemental materials.

#### 2.6.3 Determination of Cytotoxic Potential

The in vitro cytotoxicity assay was performed as a preliminary screening to assess the effects of *E. peplis* aerial‐part extracts on cell viability and to identify extracts with potential selective activity toward tumor cells for future dose‐response and mechanistic studies. The human cell lines HepG2 (hepatocellular carcinoma), SH‐SY5Y (neuroblastoma), and HEK 293 (embryonic kidney) were cultured under standard laboratory conditions, following the procedure described by Rodrigues et al. (2016).

The cytotoxicity of the plant extracts was assessed using the MTT assay. Briefly, cells were seeded in 96‐well plates at a density of 5000 cells per well and allowed to adhere overnight. Cells were then exposed to the extracts at a concentration of 100 μg/mL for 72 h, while untreated cells were used as controls. After treatment, MTT solution was added to each well, and the plates were incubated for 2 h to allow viable cells to reduce the yellow tetrazolium salt into purple formazan crystals. The formazan crystals were subsequently dissolved, and absorbance was measured using a microplate reader. Cell viability was calculated by comparing the absorbance of extract‐treated cells with that of untreated control cells, and the results were expressed as percentage of viable cells relative to the control.

### 2.7 Network Pharmacology

Network pharmacology analysis was conducted using metabolites that showed significant differences in relative abundance, as these were considered the primary contributors to the observed bioactivities. The PubChem database (https://pubchem.ncbi.nlm.nih.gov/) was used to obtain the canonical SMILES of flavonoid and phenolic acid metabolites that showed significant differences in their relative content. These data were then imported into the Similarity ensemble approach (https://sea.bkslab.org/) to predict potential molecular targets (Wang et al. 2016). SEA is a ligand‐based target identification method that predicts potential protein targets by comparing the chemical similarity of query compounds against ligand sets associated with known biological targets. The method employs molecular fingerprint‐based similarity calculations using the Tanimoto coefficient and evaluates the statistical significance of ligand‐set similarities through expectation (*E*) values. Potential targets were prioritized according to SEA *E*‐values, where lower *E*‐values indicate a lower probability that the observed chemical similarity occurred by chance. Targets with statistically significant SEA scores reported by the server were retained for subsequent analyses. SEA was selected because it enables rapid and reliable large‐scale target prediction without requiring protein structural information and has been widely applied in studies of natural products, polypharmacology, and drug repurposing (Jimenes‐Vargas et al. 2024).

Antioxidant‐related gene targets were identified through the GeneCards database (https://www.genecards.org/) (Stelzer et al. 2016). After removing duplicate targets, Venny software (version 2.1.0) was used to identify common targets between metabolite‐related and antioxidant‐related genes, resulting in a set of antioxidant‐associated targets. These targets were subsequently imported into the STRING 11.0 database (https://string‐db.org/) to obtain protein–protein interaction (PPI) data, using a confidence score threshold of 0.4 or higher. Due to limited data on protein interactions in the recently identified Camellia species, a confidence threshold of 0.4 was chosen in the STRING database to include a wider range of potential interactions. Although higher thresholds increase specificity, a lower threshold is more suitable for exploratory analysis of under‐characterized species, as it reduces the risk of missing key antioxidant targets. The resulting PPI network was visualized using Cytoscape 3.7.1 software (Cytoscape Consortium, San Diego, CA, USA).

### 2.8 GO Enrichment and KEGG Pathway Analysis

The targets underwent Gene Ontology (GO) and Kyoto Encyclopedia of Genes and Genomes (KEGG) pathway analysis using the DAVID 6.8 database (https://david.ncifcrf.gov/home.jsp), with a significance cutoff set at *p* < 0.05 (Dennis Jr et al. 2003). Based on the *p*‐values, the top ten most significant biological processes (BPs), cellular components (CCs), and molecular functions (MFs), along with the top 20 KEGG pathways, were selected for visualization using Omicshare (http://www.omicshare.com/tools/index.php/) (Mu et al. 2024). A network diagram showing the relationships among components, targets, pathways, and diseases was created using Cytoscape 3.7.1. Core protein targets were identified using the MCC algorithm in the CytoHubba plug‐in and the MCODE plug‐in within Cytoscape (Chin et al. 2014).

### 2.9 Molecular Docking

Docking calculations were performed to estimate the binding affinity of *Euphorbia peplis‐derived* phytochemicals toward selected therapeutic targets. The protein targets were selected based on their direct relevance to the experimentally validated enzyme inhibitory and antimicrobial activities (Joshi, Bachhar, Mishra, et al. 2025; Joshi, Mishra, Haldhar, et al. 2025; Joshi, Tewari, Pande, et al. 2025). Human AChE, BChE, amylase, glucosidase, and tyrosinase were included, as inhibition of these enzymes is closely linked to anti‐Alzheimer's, antidiabetic, and anti‐melanogenic effects and represents a well‐established therapeutic strategy (Colovic et al. 2013; Dirir et al. 2021). Cancer therapeutic targets were guided by DepMap‐based (https://depmap.org/portal/) vulnerability patterns in related cancer models (Shimada et al. 2021) and then refined by literature support and the availability of high‐resolution three‐dimensional structures on the protein data bank (PDB) (Berman 2000) (Table S1). Ligand structures were prepared in ChemDraw. Ligands were geometry‐optimized in Avogadro v1.2.0; polar hydrogens were added, and Gasteiger charges were assigned using AutoDockTools v4.2.6 (Cetiz et al. 2024, 2025; Morris et al. 1998). Docking was carried out with AutoDock Vina v1.1.2, with exhaustiveness set to 32 (Eberhardt et al. 2021; Trott and Olson 2010; Zengin et al. 2025). Binding pockets based on cavity predictions from the POCASA v1.1 server, and the docking grid was set (Kalyniukova et al. 2025; Yu et al. 2010). For protocol validation, co‐crystallized ligands were re‐docked into their native binding sites, and root mean square deviation (RMSD) values were computed to evaluate pose reproducibility (Ahmed et al. 2025). Protein‐ligand contact patterns such as hydrogen bonds, hydrophobic contacts, and π‐stacking interactions were examined with the Protein‐Ligand Interaction Profiler (PLIP) (Schake et al. 2025; Yagi et al. 2025).

### 2.10 Molecular Dynamics Simulation

The dynamic stability and binding behavior of selected protein–ligand complexes were assessed using molecular dynamics simulations. Initial system setup was performed with CHARMM‐GUI https://www.charmm‐gui.org/ (Jo et al. 2008). Proteins and ligands were parameterized with the CHARMM36m force field and solvated in an explicit TIP3P water box; systems were electrically neutralized by adding counterions, and the ionic strength was adjusted to 0.15 M NaCl (Jorgensen et al. 1983; Huang et al. 2017; Maier et al. 2015). Non‐bonded interactions were treated using the Verlet cutoff scheme, covalent bonds involving hydrogen atoms were constrained with LINCS, and long‐range electrostatics were computed using the particle‐mesh Ewald method. Energy minimization was carried out using the steepest descent algorithm until the maximum force fell below 1000 kJ mol^−1^ nm^−1^ (Essmann et al. 1995; Hess et al. 1997). The systems were equilibrated in two consecutive steps, first under NVT and then under NPT conditions at 310 K, followed by 100 ns production runs performed with GROMACS v2024.3 (Abraham et al. 2015; Stojković et al. 2025).

### 2.11 Statistical Analysis

All experiments were carried out in triplicate, and differences between extracts were assessed for statistical significance. First, data normality was examined using the Shapiro–Wilk test. Any descriptor with a *p*‐value greater than 0.05 was regarded as normally distributed. For these normally distributed descriptors, one‐way ANOVA, followed by Tukey's post hoc test for pairwise comparisons, was used to compare the samples. Statistical significance was defined as *p* < 0.05. The statistical and Pearson correlation analyses were performed using GraphPad Prism, version 9.2.

## 3 Results

### 3.1 Total Phenolic (TPC) and Flavonoids (TFC) Contents

Phenolic compounds are well known for their antioxidant properties. They can scavenge free radicals generated by reactive oxygen species (ROS), inhibit peroxide formation, and chelate transition metals (Zeb 2021). The TPC and TFC of different extracts of *E. peplis* were determined, and the results are presented in Table 1. The TPC ranged between 20.10 and 49.32 mg GAE/g and ranked in different extracts as follows: 70% EtOH > EtOH > Aq > EtOAc. The TFC ranged between 12.84 and 45.49 mg RE/g and ranked in different extracts as follows: EtOH > 70% EtOH > Aq > EtOAc. A previous study on the TPC and TFC of *E. tirucalli* reported that MeOH extracted a higher TPC than water, whereas the aqueous extract yielded a greater TFC (Munro et al. 2015). In contrast, the present study found that 70% EtOH was the most effective solvent for obtaining the highest TPC, while absolute EtOH produced the greatest TFC (*p* < 0.05). The TPC and TFC of *E. biumbellata, E* *. terracina* , and *E. dendroides* were evaluated, and the 70% methanol extract yielded the highest values (Zeghad et al. 2016). This finding indicates that hydroalcoholic solvents are more effective in recovering higher TPC from *Euphorbia* species.

**TABLE 1:** Extraction yields (%), total phenolic and flavonoid content in extracts of *Euphorbia peplis* aerial parts.

Extracts	Extraction yields (%)	TPC (mg GAE/g)	TFC (mg RE/g)
EtOAc	5.04	20.10 ± 0.63^d^	12.84 ± 0.04^d^
EtOH	8.80	54.78 ± 0.97^b^	45.49 ± 0.26^a^
70% EtOH	25.40	68.12 ± 2.23^a^	30.06 ± 0.33^b^
Water	21.44	49.32 ± 0.62^c^	20.72 ± 0.16^c^

*Note:* Values are reported as mean ± SD of three parallel measurements. Different letters indicate significant differences between the tested extracts (*p* < 0.05).

Abbreviations: GAE, Gallic acid equivalent; RE, Rutin equivalent.

### 3.2 Phytochemical Profile of Four Extracts From *Euphorbia peplis*

**FIGURE 1:**
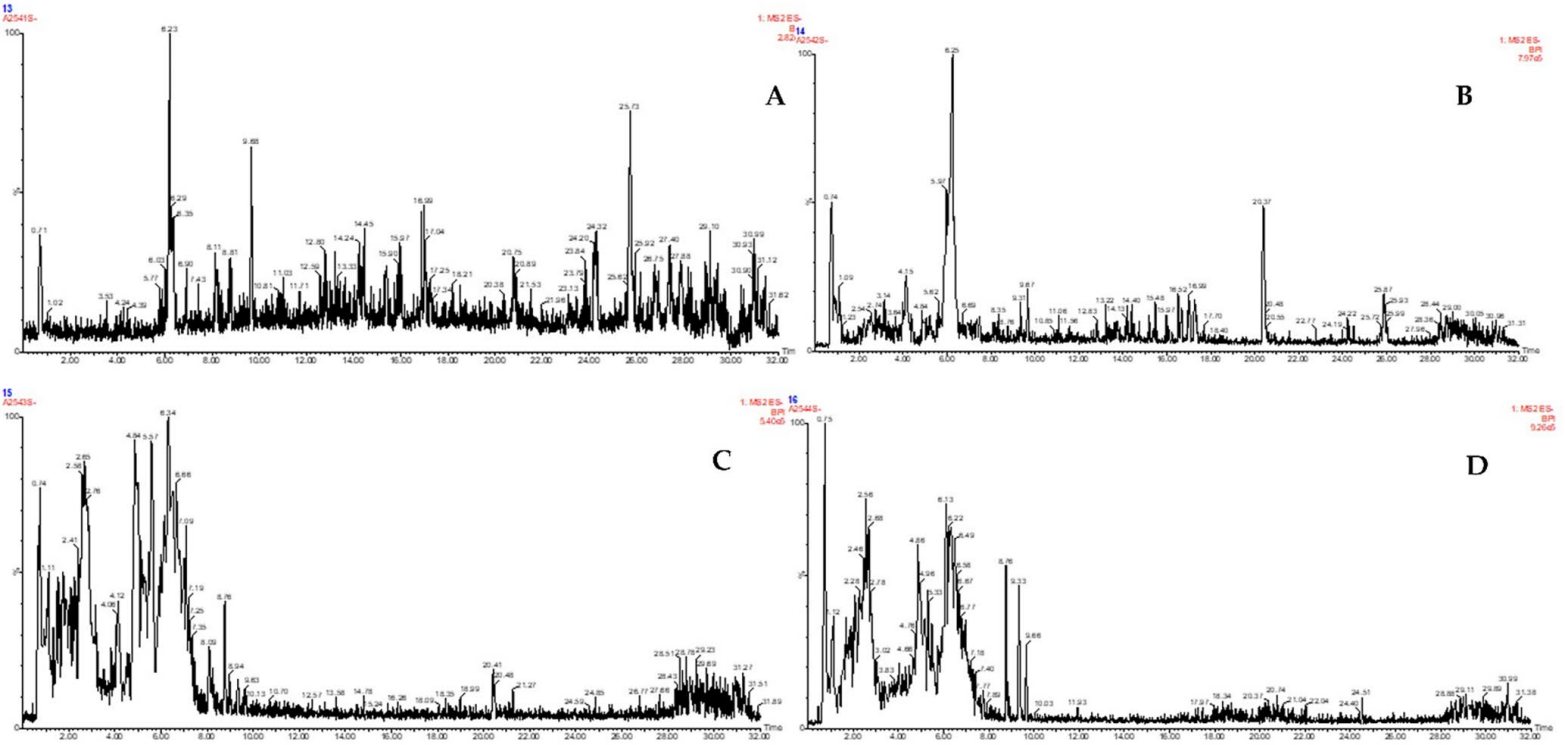
BPI chromatograms in negative ion mode for (A) Ethyl acetate, (B) Ethanol, (C) Ethanol/water and (D) Water extracts of *Euphorbia peplis* .

**FIGURE 2:**
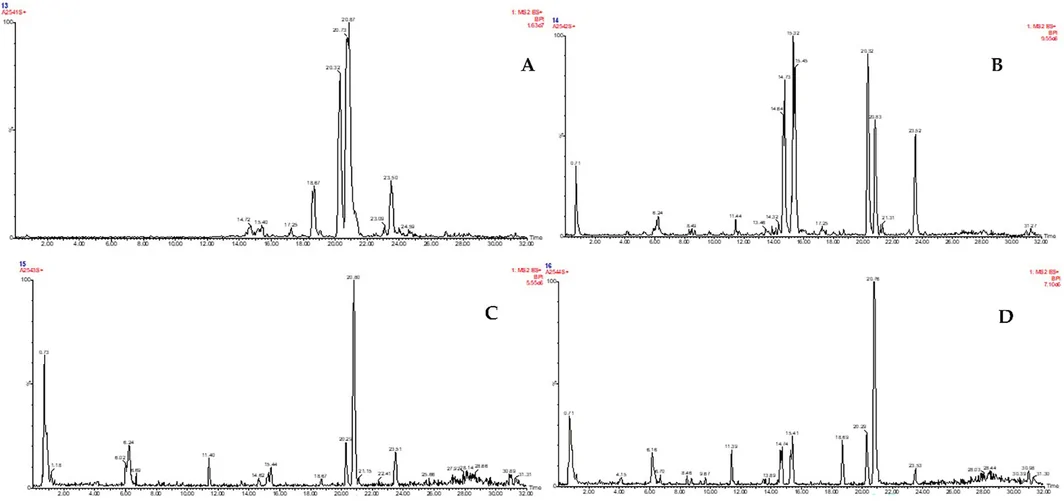
BPI chromatograms in positive ion mode for (A) ethyl acetate, (B) ethanol, (C) ethanol/water and (D) water extracts of *Euphorbia peplis* .

**FIGURE 3:**
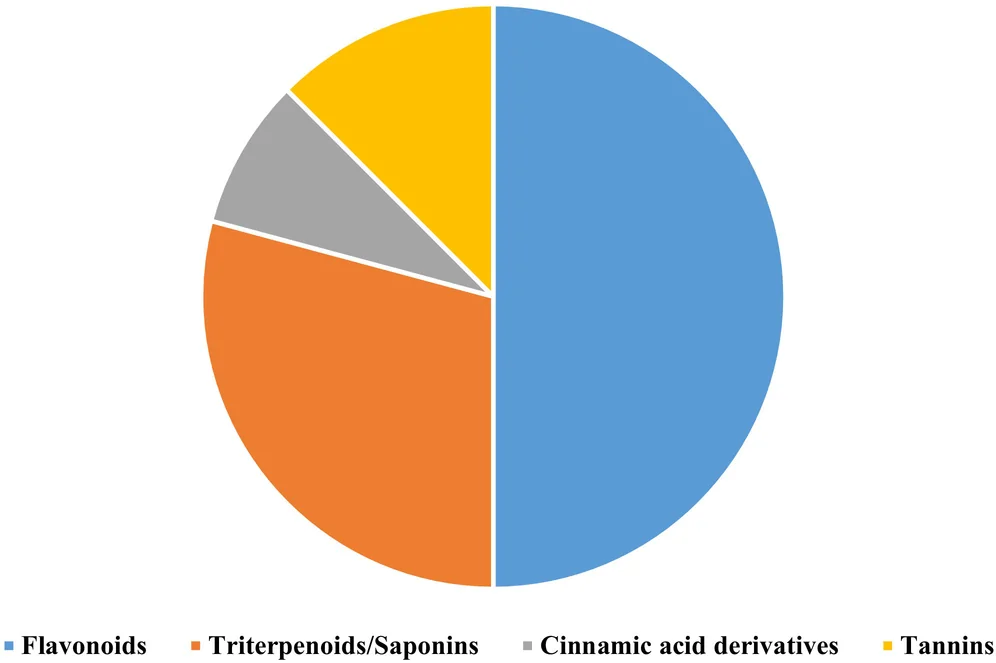
Phytochemical classes putatively identified from *Euphorbia* extracts.

In this study, comparative phytochemical profiling was employed for four extracts of *Euphorbia peplis* . UPLC/MS^n^ analysis was carried out in ESI negative and positive modes, as presented in Figures 1 and 2, respectively. Twenty‐four metabolites were tentatively identified, with flavonoids being the most abundant class. Notable variation was observed in the distribution of these compounds, with flavonoids constituting the major component in all extracts. Cinnamic acid derivatives were detected only in the EtOAc extract, while tannins were present in both the EtOAc and 70% EtOH extracts. Triterpenoids and saponins were predominantly recovered in the EtOH extract (Figure 3). The putatively annotated compounds may be detailed as follows:

#### 3.2.1 Flavonoids

Flavonoids represent a famous class of secondary metabolites found in many plant species. *Euphorbia* is well‐documented for accumulating flavonoids as one of its abundant components. Likewise, herein, 12 components were tentatively assigned to this class as flavonoid aglycones, glycosides, galloylated and hydroxylated derivatives (Table 2). Three quercetin derivatives were detected, one peak at *m/z* 463 (465) with fragments at *m/z* 431, 320, 301, 274, 225, 179 due to the loss of the quercetin aglycone (*m/z* 301) and hexose sugar (*m/z* 179). Thus, this peak was assigned to isoquercitrin (also known as quercetin‐hexoside) (de Freitas Tostes et al. 2019). Another two peaks with the same *m/z* value at 463 were observed with fragments at *m/z* 426, 363, 327, 301, 289, 226, 186, 179 sharing the main fragment at *m/z* 301 for the loss of the aglycone moiety and at *m/z* 179 for the loss of the allose sugar. These two peaks were tentatively defined as quercetin‐alloside and its isomer (de Freitas Tostes et al. 2019; Magozwi et al. 2021). Moreover, a deprotonated peak appeared at *m/z* 417 with MS/MS fragments at *m/z* 351, 301, 243, 197, 169, which was defined as kaempferol‐pentoside (Magozwi et al. 2021; Sulaiman et al. 2023; Gupta et al. 2017). In addition, one flavanone glycoside, prunin, had a deprotonated molecular ion peak at *m/z* 433 and fragments at *m/z* 391, 373, 307, 245, 197, 155 (Sulaiman et al. 2023).

**TABLE 2:** Tentatively identified metabolites from *Euphorbia peplis* extracts.

No.	Compound name	Molecular Formula	R_t_ (min.)	[M‐H]^−^ *m/z*	[M + H]^+^ *m/z*	MS/MS Fragments	% Composition				References
							EtOAc	Ethanol	70% Ethanol	Water	
*Flavonoids*											
1	Isoquercitrin (Quercetin‐hexoside)^a^	C_21_H_20_O_12_	0.71	463	465	431, 320, 274, 225, 179	√	√	√	√	de Freitas Tostes et al. (2019)
2	Isovitexin (Apigenin‐hexoside)^a^	C_21_H_20_O_10_	4.24	431	—	389, 356, 334, 303, 274, 175	√	√	√	√	Sulaiman et al. (2023)
3	Isovitexin‐hexoside (Saponarin)^a^	C_5_O_30_H_27_	25.73	593	—	347, 329, 214, 195	√	—	—	—	Ahmed et al. (2022)
4	Patuletin‐hexoside‐sulfate^a^	C_22_H_22_O_16_S	4.83	633	—	381, 338, 315, 245, 197, 171	—	—	√	√	Aljohani et al. (2022)
5	Prunin^a^	C_21_H_22_O_10_	5.63	433	—	391, 373, 307, 245, 197, 155	—	√	√	—	Sulaiman et al. (2023)
6	Quercetin‐alloside^a^	C_21_H_20_O_12_	5.77	463	—	426, 363, 327, 289, 226, 186, 169	√	√	√	√	de Freitas Tostes et al. (2019), Magozwi et al. (2021)
7	Quercetin‐alloside isomer^a^	C_21_H_20_O_12_	6.23	463	—	420, 406, 298, 293, 275, 255	√	√	√	√	de Freitas Tostes et al. (2019), Magozwi et al. (2021)
8	Kaempferol‐pentoside^a^	C_20_H_18_O_10_	6.69	417	—	351, 301, 243, 197, 169	—	√	√	—	Magozwi et al. (2021), Sulaiman et al. (2023), Gupta et al. (2017)
9	Resveratrol‐galloyl‐hexoside	C_27_H_26_O_12_	6.91	541	—	489, 399, 352, 327, 301, 297, 242, 187	√	—	—	—	Salih et al. (2017)
10	Hydroxy‐*tri*‐methoxyflavone^a^	C_18_H_16_O_6_	8.81	327	—	301, 288, 281, 232, 219	√	—	√	√	Sulaiman et al. (2023)
11	*tri*‐Hydroxy‐*di*‐methoxyflavone^a^	C_17_H_14_O_7_	9.31	329	—	285, 269, 211, 199, 167, 155	—	√	√	√	Sulaiman et al. (2023)
12	Eriodictyol^a^	C_15_H_12_O_6_	9.68	287	—	264, 241, 167, 155	√	√	√	√	Sulaiman et al. (2023)
*Triterpenoids/Saponins*											
13	Methylenecycloartanol^a^	C_31_H_52_O	11.40	—	423	405, 389, 377	—	—	√	—	Zare et al. (2015)
14	Methylenecholesterol^a^	C_28_H_46_O	13.88	—	399	351, 295, 277	—	—	—	√	Baisted et al. (1968), Giner et al. (2000)
15	*di*‐Hydroxyursenone^a^	C_30_H_46_O_3_	15.95	—	457	439, 421, 393	—	√	—	√	Warnaar (1987), Starratt (1966)
16	Cycloartenol^a^	C_30_H_50_O	21.22	—	409	393, 377, 365	—	√	—	—	Tavčar et al. (2012)
17	Friedelin^a^	C_30_H_50_O	23.49	—	441	425, 409, 392, 285, 277	√	√	—	—	Yener et al. (2018)
18	Euphol^a^	C_30_H_50_O	25.92	—	427	409, 393, 365, 339	—	√	—	—	Benjamaa et al. (2022), Dutra et al. (2011), Cruz et al. (2018)
19	Gypsogenin^a^	C_30_H_46_O	17.24	469	—	421, 396, 356, 313, 287, 230, 197, 181	√	√	—	—	Choene and Motadi (2016)
*Cinnamic acid derivatives*											
20	*di*‐Caffeoylquinic acid	C_25_H_24_O_12_	4.42	515	—	421, 379, 293, 231, 212, 179, 155	√	—	—	—	Attia et al. (2022)
21	Chlorogenic acid^a^	C_16_H_18_O_9_	17.51	353	—	328, 311, 297, 270, 250, 177, 166	√	—	—	—	Saleh et al. (2023), Zhang et al. (2017), Smeriglio et al. (2021)
*Tannins*											
22	Ellagic acid^a^	C_14_H_6_O_8_	8.16	301	303	283, 257, 236, 215, 191, 181	√	—	√	—	Lan et al. (2020), Bindra et al. (1988)
23	*tri*‐Galloyl‐hexose^a^	C_27_H_24_O_18_	21.96	635	—	584, 528, 485, 393, 353, 293, 281, 269, 181	√	—	—	—	Mekam et al. (2019)
24	*di*‐Galloyl‐hexose^a^	C_20_H_20_O_14_	30.97	483	—	419, 385, 353, 313, 311, 258, 196, 183, 181	√	—	—	—	Sulaiman et al. (2023)
No. of identified compounds							15	14	12	10	

Abbreviations: EtOAc, ethyl aceate; EtOH, ethanol; Ref, references.

^a^ For metabolites detected before from genus *Euphorbia*.

Two apigenin derivatives appeared at *m/z* 431 and 593 with fragments at *m/z* 389, 356, 334, 303, 274, 175 and *m/z* 347, 329, 214, 195, respectively. Thus, they were tentatively identified as isovitexin (also known as apigenin‐hexoside) and isovitexin‐hexoside (also known as saponarin), respectively. Different other flavonoids were detected and listed in Table 2 with their parent peak and respective fragments like patuletin‐hexoside‐sulfate (Aljohani et al. 2022), resveratrol‐galloyl‐hexoside (Salih et al. 2017), hydroxy‐*tri*‐methoxyflavone (Sulaiman et al. 2023), *tri*‐hydroxy‐*di*‐methoxyflavone (Sulaiman et al. 2023), and eriodictyol (Sulaiman et al. 2023).

#### 3.2.2 Triterpenoids/Saponins

Triterpenoids and saponins were recorded as the second most abundant class after flavonoids in our study (Table 2). Most of them were ionized in the ESI positive mode. In this regard, a protonated molecular ion peak was detected at *m/z* 423 with fragments at *m/z* 405, 389, 377 due to loss of methyl group followed by cleavage of the triterpenoid ring thus it was tentatively identified as methylene‐cycloartanol (Zare et al. 2015). Moreover, cycloartenol was observed at *m/z* 409 and it gave rise to fragments at *m/z* 393, 377, 365 (Tavčar et al. 2012). Similarly, methylene‐cholesterol presented a parent peak at *m/z* 399 with daughter fragments at *m/z* 351, 295, 277 (Baisted et al. 1968; Giner et al. 2000). In addition, a parent peak was recorded at *m/z* 457 with MS/MS fragments at *m/z* 439, 421, 393 due to the loss of a hydroxyl group, followed by the successive fragmentation and cleavage of the ursane ring thus, this peak was tentatively assigned to *di*‐hydroxyursenone (Warnaar 1987; Starratt 1966). Three more components were also detected, including friedelin (*m/z* 441) (Yener et al. 2018), euphol (*m/z* 427) (Benjamaa et al. 2022; Dutra et al. 2011; Cruz et al. 2018) and gypsogenin (*m/z* 469, ESI ‐ve mode) (Choene and Motadi 2016).

#### 3.2.3 Cinnamic Acid Derivatives

Two cinnamic acid derivatives were tentatively identified in our study. They presented their deprotonated peaks at *m/z* 515 and 353 with fragments at *m/z* 421, 379, 293, 231, 212, 179, 155 and *m/z* 328, 311, 297, 270, 250, 177, 166, respectively. Thus, they were assigned to *di*‐caffeoylquinic acid (Attia et al. 2022) and chlorogenic acid (Saleh et al. 2023; Zhang et al. 2017; Smeriglio et al. 2021), respectively (Table 2). It is worth noting that the peak at *m/z* in the first compound's fragmentation is confirmatory for the presence of caffeic acid in its structure.

#### 3.2.4 Tannins

Three tannins, previously reported from genus *Euphorbia*, were detected herein, including ellagic acid (Lan et al. 2020; Bindra et al. 1988), *tri*‐galloyl‐hexose (Mekam et al. 2019) and *di*‐galloyl‐hexose (Sulaiman et al. 2023) their deprotonated peaks appeared at *m/z* 301 (303), 635 and 483, respectively. All of these tannins were traced from the ethyl acetate extract (Table 2).

### 3.3 Multivariate Data Analysis Using PCA and Clustered Heat Map

**FIGURE 4:**
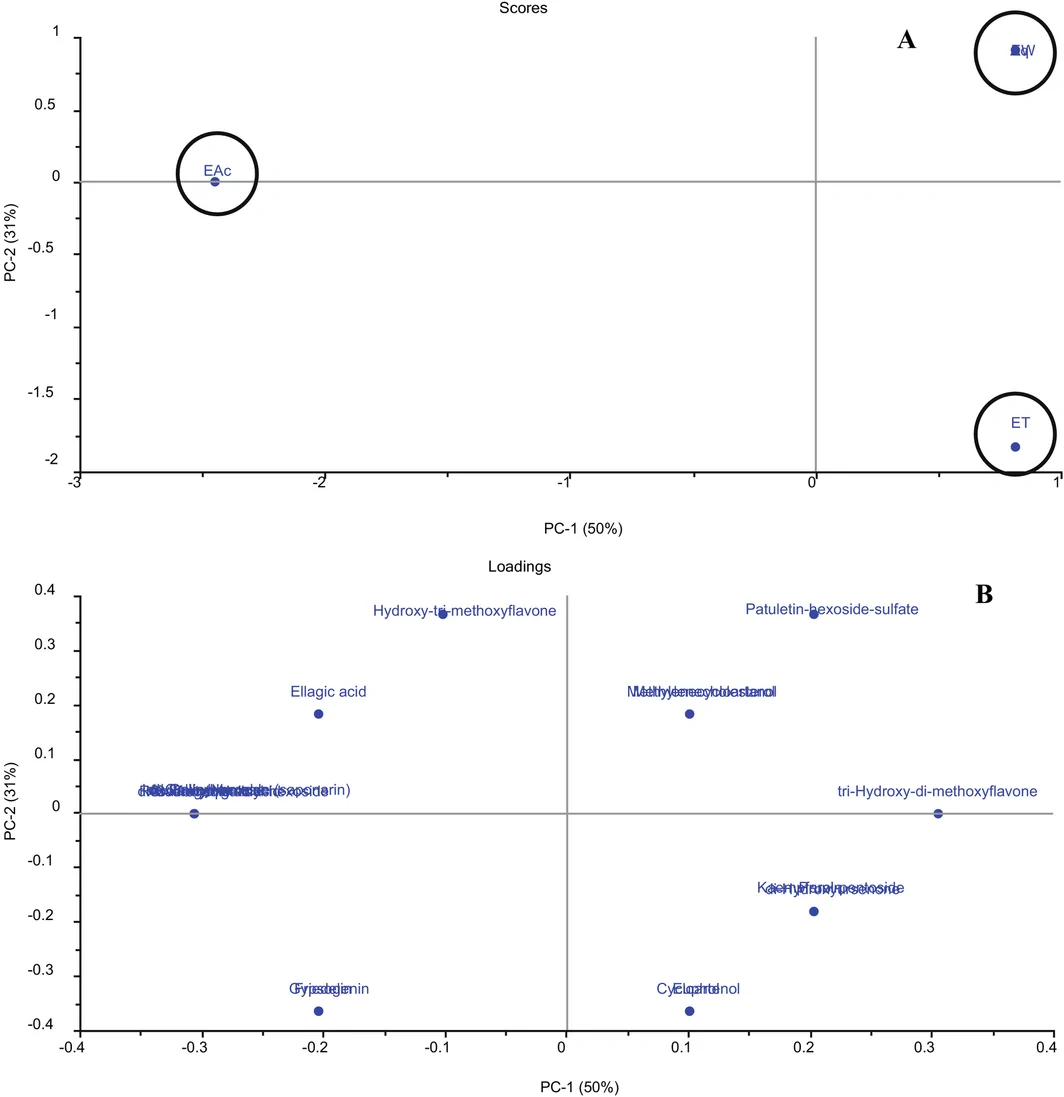
PCA plots presented as (A) Score plot of PC1 versus PC2 of the tentatively identified components from *Euphorbia peplis* extracts (component as a variable). (B) Loading plot for PC1 and PC2 contributing metabolites and their assignments (component as variable). Aq, aqueous; EAc, ethyl aceate; ET, ethanol; EW, ethanol/water.

**FIGURE 5:**
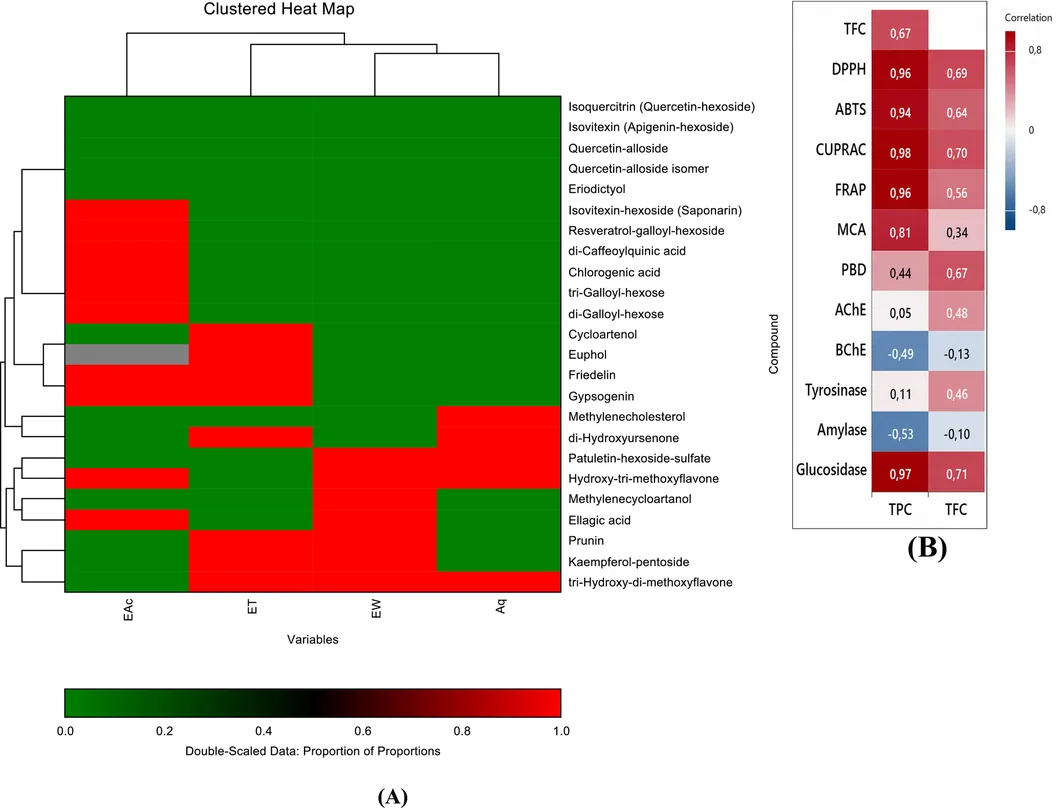
(A) Clustered heat map showing the tentatively identified metabolites from *Euphorbia peplis* extracts. A heat map was constructed using Euclidean distance and the unweighted group method. Aq, aqueous; EAc, ethyl acetate; ET, ethanol; EW, ethanol/water. (B) Pearson correlation between total phenolic (TPC)/total flavonoid content (TFC) and biological activities.

Multivariate data analysis was performed through the unsupervised technique, principal component analysis (PCA), and a clustered heat map for discrimination between the four analyzed extracts. The PCA analysis resulted in three distinct clusters with PC1 (50%) and PC2 (31%). The clusters appeared as one for the ethyl acetate extract (EtOAc), another for the ethanol extract (EtOH), while the third cluster showed an overlap between the 70% ethanol and the aqueous extracts (Aq) (Figure 4A). The overlap in the third cluster could be explained by the presence of certain common metabolites between 70% ethanol and Aq viz. patuletin‐hexoside‐sulfate, as shown in the PCA loading plot (Figure 4B). In addition, the presence of certain components like isovitexin‐hexoside (saponarin), resveratrol‐galloyl‐hexoside, *di*‐caffeoylquinic acid, chlorogenic acid, *tri*‐galloyl‐hexose and *di*‐galloyl‐hexose only in the EtOAc extract (the overlapping components in the PCA loading plot) contributed to its distinct clustering pattern. Likewise, the ET extract showed the unique presence of cycloartenol and euphol, thus located as one cluster. The clustered heat map showed the same clustering pattern as observed in the PCA, with visualization of data by color (Figure 5).

This genus is marked by the presence of flavonoids, *di*‐ and triterpenoids, which came in line with the classes traced herein our study. Several studies have reported the presence of several classes of terpenoids, which are regarded as important chemotaxonomic markers of the genus *Euphorbia* and are frequently associated with significant biological activities, including cytotoxic and anti‐inflammatory effects. In addition, flavonoids and other polyphenolic constituents have been putatively annotated, contributing to the antioxidant potential of the plant. The coexistence of these bioactive classes supports the traditional medicinal relevance of *E. peplis* and highlights its potential as a source of pharmacologically valuable natural products (Kemboi et al. 2020). *Euphorbia* species are well‐known to accumulate phenolic compounds and flavonoids. For instance, the ethyl acetate, methanol, and aqueous extracts of *E. hirta* , *E. heterophylla* , and *E. convolvuloides* were profiled via LC‐DAD‐MS^n^ and flavonoid glycosides, phenolic acids, etc., were detected, along with the determination of their total phenolic/flavonoid contents (Mahomoodally et al. 2020). Similarly, *E. milii* ethyl acetate fraction was profiled through LC‐ESI‐TOF‐MS and a diverse phenolic/flavonoid composition was revealed and linked with its antimicrobial effects (Hassan et al. 2023). Moreover, a comparative phenolic profiling among *Euphorbia* species in Iraq (including *E. peplus* ) found nine common phenolic compounds (gallic acid, caffeic acid, quercetin, kaempferol and myricetin) (Taib et al. 2022).

### 3.4 Biological Investigations of the Aerial Parts Extracts of *Euphorbia peplis*

#### 3.4.1 Antioxidant Activity

Oxidative stress has been purported as one of the mechanisms associated with oxidative damage caused by free radicals and leading to chronic pathologies like cancer, coronary heart disease, diabetes, neurodegenerative diseases and many others (Pooja et al. 2025). Antioxidants can counteract the harmful effects of reactive oxygen species (ROS), which lead to oxidative stress. The antioxidant activity of different extracts of *E. peplis* was determined using various assays. DPPH and ATBS assays measure the ability of the extract to scavenge free radicals and FRAP and CUPRAC assays indicate the reducing capacity of the extract. The phosphomolybdenum (PBD) assay evaluates its effectiveness in reducing the Mo (VI) to (MoV) while the metal chelating ability (MCA) of the extract is determined by measuring its iron chelating ability. Results are presented in Table 3. Globally, the three polar extracts exerted remarkable antioxidant activity, suggesting the polar nature of antioxidant molecules. The 70% EtOH extract showed the highest significant antiradical (DPPH = 385.50 mg TE/g; ABTS = 466.31 mg TE/g, *p* < 0.05) and ions reducing (CUPRAC = 439.95 mg TE/g; FRAP = 305.07 mg TE/g, *p* < 0.05) activities and, together with the aqueous and EtOH extracts, demonstrated the greatest metal‐chelating (19.39 and 20.60 mg EDTAE/g; *p* ≥ 0.05) and total antioxidant activities (2.45 and 2.43 mmol TE/g; *p* ≥ 0.05) respectively.

**TABLE 3:** Antioxidant activity of extracts of *Euphorbia peplis* aerial parts.

Extracts	DPPH (mg TE/g)	ABTS (mg TE/g)	CUPRAC (mg TE/g)	FRAP (mg TE/g)	MCA (mg EDTAE/g)	PBD (mmol TE/g)
EtOAc	30.38 ± 1.01^d^	38.44 ± 0.80^d^	89.02 ± 2.78^d^	42.86 ± 0.30^d^	12.92 ± 1.47^c^	2.08 ± 0.03^b^
EtOH	349.56 ± 8.41^b^	417.32 ± 1.92^c^	380.24 ± 7.52^b^	229.58 ± 2.23^c^	17.37 ± 0.22^b^	2.43 ± 0.06^a^
70% EtOH	385.50 ± 2.23^a^	466.31 ± 1.78^a^	439.95 ± 7.61^a^	305.07 ± 2.73^a^	19.39 ± 0.33^a^	2.45 ± 0.01^a^
Aqueous	335.89 ± 1.88^c^	436.85 ± 2.77^b^	351.22 ± 7.47^c^	260.25 ± 1.17^b^	20.60 ± 0.21^a^	1.72 ± 0.01^c^

*Note:* Values are reported as mean ± SD of three parallel measurements. Different letters indicate significant differences between the tested extracts (*p* < 0.05).

Abbreviations: EDTAE, EDTA equivalent; MCA, Metal chelating activity; PBD, Phosphomolybdenum; TE, Trolox equivalent.

The EtOH extract showed the second‐highest activity in the DPPH, CUPRAC, and MCA assays, while the aqueous extract ranked second in the ABTS and FRAP assays. The EtOAc extract exerted the least antioxidant activity in all assays except the PBD where it displayed the second best. The results were consistent with the total phenolic content of the extracts, showing that the 70% EtOH extract possessed the highest antioxidant activity and TPC. Pearson correlation analysis also confirmed this, revealing a strong correlation (*R* > 0.8) between TPC and antioxidant effects (except for PBD) (Figure 5B). Moreover, HPLC‐ESI‐Q‐TOF‐MS analysis revealed that the 70% EtOH extract contained the greatest number of identified flavonoid compounds. Notably, derivatives of quercetin, apigenin, kaempferol, prunin, and eriodictyol are well known for their antioxidant properties (Deng et al. 2020; Zhang and Ye 2020; Kashyap et al. 2022; Septembre‐Malaterre et al. 2022). When these findings were compared with those reported for *E. denticulata* by Gokhan et al. (Zengin et al. 2017), *E. peplis* exhibited significantly stronger antioxidant activity in all assays except metal‐chelating capacity. In addition to species‐specific differences in biological properties, the extraction method and solvent type play crucial roles in the recovery of bioactive compounds. Overall, our results indicated that 70% ethanol was the most effective solvent. Other *Euphorbia* species, including *E. hylonoma, E. inaequilatera, E. milii, E. biumbellata, E. terracina*, *E. tirucalli* and *E. dendroides* were evaluated for their antiradical activity and results showed that they exhibited notable antiradical activity, and hence the genus could be a promising source of antioxidant molecules (Munro et al. 2015; Zeghad et al. 2016; Ernst et al. 2015; Chohan et al. 2020).

#### 3.4.2 Enzyme Inhibition Activity

Inhibition of enzymes is one of the key strategies for treating a wide range of diseases, including Alzheimer's disease, skin pigmentation disorders, and diabetes (Naz et al. 2022). Naturally occurring compounds from plants are a potential source of new inhibitors. The enzyme inhibitory activity of different extracts of *E. peplis* was determined against AChE, BChE, tyrosinase, *α*‐amylase and *α*‐glucosidase enzymes, and the results are presented in Table 4. Across most assays, the three organic extracts demonstrated effectiveness with differing degrees of activity, while the aqueous extract was generally ineffective or displayed only low activity. This trend suggests that the major enzyme‐inhibiting constituents of *E. peplis* are predominantly medium to low‐polarity phytochemicals that are more efficiently extracted by organic solvents than by water. The EtOH extract showed the highest anti‐AChE activity (2.84 mg GALAE/g), while the 70% EtOH and EtOAc extracts exhibited comparable activities (2.42 and 2.26 mg GALAE/g; *p* ≥ 0.05). For BChE inhibition, the EtOAc extract was the most effective (2.18 mg GALAE/g, *p* < 0.05), with the EtOH and 70% EtOH extracts displaying similar inhibitory effects (1.26 and 1.24 mg GALAE/g; *p* ≥ 0.05). The differential inhibition of AChE and BChE among extracts indicates selective enzyme inhibition behavior, likely arising from differences in phytochemical composition. Since BChE possesses a larger and more hydrophobic active‐site gorge than AChE, less polar constituents enriched in the EtOAc extract may interact more favorably with this enzyme, accounting for its superior BChE inhibitory activity (Rosenberry et al. 2017). Conversely, the broader phytochemical profile of the ethanolic extract, including polar phenolics and flavonoids, may contribute to stronger AChE inhibition through multiple binding interactions, including hydrogen bonding and π–π stacking with aromatic residues within the catalytic gorge (Khan et al. 2018; Roseiro et al. 2012). Tyrosinase inhibition followed a different pattern, with the EtOH and 70% EtOH extracts exhibiting the highest activities (54.40 and 53.39 mg KAE/g; *p* ≥ 0.05) followed by the EtOAc extract (49.33 mg KAE/g). This result suggests that relatively polar phenolic compounds are important contributors to tyrosinase inhibition. The mechanism of tyrosinase inhibition is frequently associated with the ability of phenolics and flavonoids to chelate the copper ions located at the enzyme active site and/or compete with the natural substrate through interactions involving hydroxyl groups (Zolghadri et al. 2019). Therefore, the elevated tyrosinase inhibitory activity of the hydroethanolic and ethanolic extracts may be linked to their higher abundance of phenolic constituents.

**TABLE 4:** Enzyme inhibitory activity of extracts of *Euphorbia peplis* aerial parts.

Extracts	AChE (mg GALAE/g)	BChE (mg GALAE/g)	Tyrosinase (mg KAE/g)	Amylase (mmol ACAE/g)	Glucosidase (mmol ACAE/g)
EtOAc	2.26 ± 0.18^b^	2.18 ± 0.38^a^	49.33 ± 1.32^b^	0.50 ± 0.03^a^	na
EtOH	2.84 ± 0.01^a^	1.26 ± 0.06^b^	54.40 ± 0.45^a^	0.34 ± 0.01^b^	1.19 ± 0.02^c^
70% EtOH	2.42 ± 0.02^b^	1.24 ± 0.16^b^	53.39 ± 0.33^a^	0.30 ± 0.01^c^	1.35 ± 0.01^a^
Aqueous	na	na	27.01 ± 1.01^c^	0.09 ± 0.01^d^	1.08 ± 0.03^d^

*Note:* Values are reported as mean ± SD of three parallel measurements. Different letters indicate significant differences between the tested extracts (*p* < 0.05).

Abbreviations: ACAE, Acarbose equivalent; GALAE, Galantamine equivalent; KAE, Kojic acid equivalent; na, not active.

Concerning the two enzymes associated with the management of diabetes, extracts were more effective toward the α‐glucosidase than the α‐amylase, with the highest inhibitory activity obtained from the 70% EtOH extract toward the former (1.35 mmol ACAE/g) and from the EtOAc extract toward the latter (0.50 mmol ACAE/g). This selectivity toward α‐glucosidase is pharmacologically relevant because potent α‐glucosidase inhibition combined with moderate α‐amylase inhibition may reduce postprandial hyperglycemia while minimizing gastrointestinal side effects commonly associated with excessive α‐amylase inhibition (Cho et al. 2011). The superior α‐glucosidase inhibition of the 70% EtOH extract may be attributed to its enrichment in flavonoids and phenolic acids, which are known to bind to catalytic residues and alter enzyme conformation (Şöhretoğlu and Sari 2020). In Pearson correlation (Figure 5B), a strong correlation between total phenolic and glucosidase inhibition was also observed (R: 0.97). The present study also the inhibitory effect of different extracts of *E. peplis* on the CAI and CAII isoenzymes of human erythrocytes. Results are presented in Table 5. In general, the extracts showed limited effectiveness toward the two isoenzymes, with inhibitory activity rising as extract polarity decreased.

**TABLE 5:** Inhibitory effects of *Euphorbia peplis* extracts on human carbonic anhydrase isoenzymes I and II (CA I and CA II).

Extracts	CA I		CA II	
	IC_50_ (μg/mL)	*R* ^2^	IC_50_ (μg/mL)	*R* ^2^
EtOAc	41.49 ± 2.49^b^	0.9401	45.00 ± 2.38^b^	0.9472
EtOH	48.46 ± 2.14^c^	0.9559	106.61 ± 2.01^c^	0.9553
70% EtOH	110.00 ± 4.52^d^	0.9589	123.75 ± 2.98^d^	0.9337
Aqueous	113.60 ± 4.62^d^	0.9593	121.57 ± 1.42^d^	0.9685
Asetazolamid (Standart inhibitor)	4.23 ± 0.07^a^	0.9836	4.81 ± 0.27^a^	0.9940

*Note:* Values are reported as mean ± SD of three parallel measurements. Different letters indicate significant differences between the tested extracts (*p* < 0.05).

Studies on the enzyme inhibitory properties of *Euphorbia* species remain limited. A novel triterpenoid, eupulcherol A, isolated from *E. pulcherrima* , was reported to exhibit anti‐Alzheimer‐related bioactivity (Yu et al. 2020). Similarly, Zengin et al. (Zengin et al. 2017) demonstrated that methanolic flower and leaf extracts of *E. denticulata* showed remarkable α‐glucosidase (10.59 and 8.18 mmol ACAE/g) and tyrosinase inhibitory activities (70.11 and 60.12 mg KAE/g), whereas their anti‐AChE and anti‐BChE activities were lower than those observed for *E. peplis* in the present study. Several metabolites identified in the current investigation have previously been associated with enzyme inhibition and may contribute to the observed bioactivities. Chlorogenic acid has been reported to inhibit AChE, tyrosinase, α‐amylase, and α‐glucosidase through interactions with catalytic residues and modulation of enzyme conformation (Oboh et al. 2015; Nguyen et al. 2024). Eriodictyol has demonstrated significant α‐glucosidase inhibitory activity (Kuroda et al. 2012). while gypsogenin has been identified as a potent AChE inhibitor (Heller et al. 2014). Furthermore, quercetin and kaempferol derivatives are well‐recognized enzyme inhibitors whose activity is influenced by the number and position of hydroxyl groups, glycosylation patterns, and overall molecular planarity, factors that govern their binding affinity toward enzyme active sites (Tiwari et al. 2023). Overall, these findings highlight *E. peplis* as a promising natural source of multifunctional enzyme inhibitors with potential applications in the management of neurodegenerative disorders, diabetes, and hyperpigmentation‐related conditions.

#### 3.4.3 Cytotoxicity Evaluation

The cytotoxic potential of *E. peplis* extracts was evaluated against three cell lines, and the results are presented in Table 6. Overall, the extracts showed marked differences in cytotoxicity and selectivity, depending on the extraction solvent. In general, the less polar and medium‐polarity extracts, particularly ethyl acetate and ethanol, displayed the strongest cytotoxic effects, whereas the aqueous and 70% ethanol extracts showed a more moderate and selective profile. This solvent‐dependent pattern is consistent with the chemical composition of the extracts, since less polar organic solvents are more efficient in recovering lipophilic and medium‐polar specialized metabolites, including triterpenoids and diterpenoids commonly reported in the genus *Euphorbia* (El‐Hawary et al. 2020; Yang et al. 2011).

**TABLE 6:** Cytotoxicity of *Euphorbia peplis* extracts on HEK 293, HepG_2_ and SH‐SY5Y cell lines, and selectivity index.

Extract	% Cellular viability			Selectivity index	
	HEK 293	HepG2	SH‐SY5Y	HEK 293/HepG2	HEK 293/SHSY5Y
EtOAc	8.18 ± 0.56^bD^	28.2 ± 2.93^aC^	4.31 ± 0.15^cD^	0.29	1.90
Ethanol	20.3 ± 1.58^bC^	28.9 ± 2.75^aC^	18.1 ± 1.23^cC^	0.70	1.12
70% Ethanol	99.7 ± 1.87^aB^	38.2 ± 4.04^cB^	71.3 ± 1.76^bB^	2.61	1.40
Water	111 ± 3.35^aA^	56.0 ± 4.13^cA^	93.3 ± 3.63^bA^	1.98	1.19

*Note:* Values are reported as mean ± standard deviation (SD) of at least three experiments performed in triplicate (*n* = 9). Different lowercase superscript letters within the same row indicate significant differences among cell lines for the same extract. Different uppercase superscript letters within the same column indicate significant differences among extracts for the same cell line. Statistical analysis was performed using one‐way ANOVA followed by pairwise comparisons based on summary statistics; *p* < 0.05 was considered statistically significant.

The ethyl acetate and ethanol extracts induced a generalized and non‐selective cytotoxic response, reducing cell viability to below 30% in all tested cell lines (Table 6). The ethyl acetate extract was particularly toxic, decreasing the viability of SH‐SY5Y neuroblastoma cells to 4.31% (*p* < 0.05) and that of non‐tumoral HEK 293 cells to 8.18% (*p* < 0.05). This strong activity may be associated with the distinctive phytochemical profile of this extract, as revealed by PCA and clustered heat map analysis, where the ethyl acetate extract formed an isolated cluster. Its separation was mainly driven by the presence of compounds detected exclusively or predominantly in this extract, including isovitexin‐hexoside (saponarin), resveratrol‐galloyl‐hexoside, di‐caffeoylquinic acid, chlorogenic acid, tri‐galloyl‐hexose and di‐galloyl‐hexose. Although several of these phenolic derivatives are frequently associated with bioactive properties, their concentration and combination with other less polar constituents may contribute to the pronounced cytotoxicity observed. Moreover, the genus *Euphorbia* is known to contain biologically active diterpenoids, including irritant compounds such as phorbol ester‐related structures, which may induce strong cellular damage independently of the tumoral or non‐tumoral origin of the cells (El‐Hawary et al. 2020). This may explain the lack of selectivity observed for the ethyl acetate extract.

A similar trend was observed for the ethanol extract, which also strongly reduced cell viability in all cell lines. The distinct clustering of this extract in the PCA and heat map analyses was associated with the unique presence of cycloartenol and euphol. These triterpenoid‐type compounds are lipophilic metabolites that are more readily extracted with organic solvents and have been associated with relevant biological activities, including cytotoxic and antiproliferative effects. Therefore, the high cytotoxicity of the ethanol extract may be related, at least in part, to the enrichment of these less polar terpenoid constituents. However, as observed for the ethyl acetate extract, this activity was not selective toward tumor cells, suggesting that these extracts may contain compounds or compound mixtures with broad cytotoxic effects.

In contrast, the more polar extracts displayed a more nuanced and potentially more relevant cytotoxic profile. The hydroethanolic extract emerged as the most promising extract for selective applications, preserving the viability of non‐tumoral HEK 293 cells at 99.7%, while still exerting a marked cytotoxic effect against HepG2 hepatocarcinoma cells, whose viability was reduced to 38.2%. Although the aqueous extract was the least cytotoxic overall, maintaining high viability in HEK 293 cells (111%) and SH‐SY5Y cells (93.3%), it still showed moderate inhibitory activity against HepG2 cells, reducing viability to 56.0%. The closer chemical similarity between the hydroethanolic and aqueous extracts, evidenced by their overlap in the PCA and confirmed by the clustered heat map, may be explained by the presence of shared polar metabolites, such as patuletin‐hexoside‐sulfate. These compounds may contribute to a more selective bioactivity profile, with lower toxicity toward non‐tumoral cells.

The selectivity index (SI), which reflects the cytotoxic selectivity of a crude extract toward cancer cells in comparison with cells of non‐tumoral origin (Prayong et al. 2007), was calculated to identify the extracts with the greatest potential for further cytotoxicity‐guided studies. An SI value higher than 3.0 is generally considered indicative of high selectivity (Machana et al. 2011). In the present study, the 70% ethanol extract showed the highest selectivity toward HepG2 cells, with an SI of 2.61, followed by the aqueous extract, with an SI of 1.98. Although these values do not reach the threshold for high selectivity, they suggest a more favorable safety profile than that of the less polar extracts. Conversely, the ethyl acetate extract showed an unfavorable SI value for HepG2 cells (SI = 0.29), confirming that its toxicity was greater toward non‐tumoral HEK 293 cells than toward tumor cells.

The pattern observed for *E. peplis* , with stronger but non‐selective cytotoxicity in less polar and medium‐polarity extracts and a more favorable selectivity profile in hydroethanolic and aqueous extracts, is consistent with previous reports on *E. peplus* and other *Euphorbia* species. For example, an ethanolic leaf extract of *E. peplus* inhibited HepG2 cell proliferation, whereas *E. peplus* ‐fabricated AuNPs produced marked inhibition of both HepG2 and HeLa cells but were also associated with complete hemolysis and dose‐dependent inhibition of splenic cells, indicating relevant systemic toxicity at higher exposures (Ghramh et al. 2019). Likewise, an aqueous extract of *E. peplus* showed strong growth inhibition of MCF‐7 breast cancer cells and induced predominantly apoptotic cell death, with ultrastructural features such as apoptotic blebbing, chromatin condensation and apoptotic bodies (Al‐Emam et al. 2019).

More recent work on an ethyl acetate extract of *E. peplus* further confirmed a clear dose‐ and time‐dependent cytotoxic effect against prostate cancer cells, with an IC_50_ of 162.5 μg/mL, supporting the anticancer potential of medium‐polarity fractions from this genus (Hasan and Abdul‐Jalil 2026). In addition, detailed phytochemical analysis of the medium‐polar ethanol fraction of *E. peplus* identified 32 diterpenoids, including compounds with measurable cytotoxicity and several ingenane‐type diterpenoids with strong anti‐inflammatory activity (Li et al. 2024). Together, these findings provide a chemical and biological rationale for the strong cytotoxicity observed in the ethyl acetate and absolute ethanol extracts of *E. peplis* . However, because these extracts also markedly affected non‐tumoral HEK 293 cells, their activity appears to be mainly non‐selective. Therefore, the hydroethanolic and aqueous extracts, particularly the 70% ethanol extract, should be prioritized in future studies aimed at identifying cytotoxic compounds with improved selectivity toward tumor cells. Further dose‐response assays and bioactivity‐guided fractionation will be required to confirm this preliminary screening and to determine whether the observed HepG2 selectivity can be linked to specific polar or moderately polar constituents.

It is important to note that this work represents a preliminary screening study, conducted at a fixed concentration of 100 μg/mL. Consequently, IC_50_ values were not calculated at this stage, as the primary goal was to identify candidate extracts for future bioguided fractionation. Based on these findings, the 70% ethanol extract could be considered an interesting candidate for future isolation and characterization of bioactive compounds, since it presented the highest SI. This selectivity suggests that the hydroethanolic mixture selectively extracts metabolites, potentially specific flavonoids or cinnamic acid derivatives identified in the chemical profiling, capable of targeting hepatocarcinoma without compromising non‐tumoral cell integrity (El‐Hawary et al. 2020; Al‐Emam et al. 2019). Future studies will focus on establishing dose‐response relationships and determining IC_50_ values, followed by the bioassay‐guided isolation and characterization of the compounds responsible for the observed cytotoxic effects, as outlined in the literature for other species (Joshi, Bachhar, Haldhar, et al. 2025). In addition, the molecular mechanisms underlying their antitumoral activity, particularly the induction of apoptosis and other cell death pathways, will be investigated.

### 3.5 Network Pharmacology Analysis of Metabolites in *Euphorbia peplis*

Potential targets were predicted using SEA, while antioxidant‐related genes were obtained from GeneCards based on the significant antioxidant activity observed experimentally. The overlapping targets were analyzed to identify key proteins and pathways. Enrichment analysis revealed involvement in oxidative stress, apoptosis, inflammation, and cancer‐related pathways. This is biologically relevant, as oxidative stress is closely associated with carcinogenesis; therefore, the identified cancer pathways were interpreted as downstream processes linked to the antioxidant effects of the metabolites.

**FIGURE 6:**
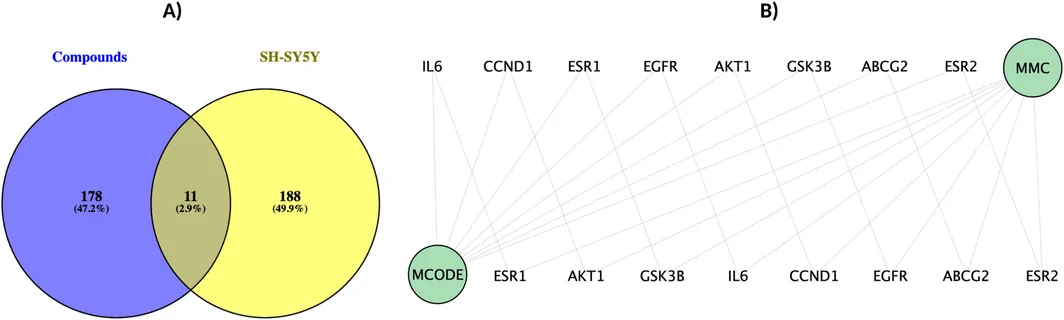
Network pharmacology analysis of compounds in *Euphorbia peplis* ‐disease targets. (A) Venn diagram showing shared targets between compounds and disease. (B) PPI network of overlapping targets highlighting hub genes and the MCODE cluster.

The top 24 metabolites in *Euphorbia peplis* were screened and compared with the PubChem database to identify compounds with SMILES numbers and corresponding antioxidant targets. A Venn diagram illustrating the overlap between compound‐associated targets and disease‐related targets is presented in Figure 6A. A total of 178 targets is uniquely associated with the identified compounds, while 188 targets are specific to the disease dataset. Importantly, 11 targets are shared between both sets, representing the putative therapeutic targets through which the compounds may exert their biological effects. Such intersection analysis is a common first step in network pharmacology, as it helps narrow down biologically relevant targets that are simultaneously modulated by bioactive compounds and implicated in disease pathophysiology (Hopkins 2008).

A PPI network was constructed from the overlapping targets, followed by topological analysis to identify hub genes (Figure 6B). Nodes such as IL6, CCND1, EGFR, AKT1, GSK3B, ESR1, ESR2, and ABCG2 exhibit high connectivity, indicating their central regulatory roles within the network. The highlighted MCODE identified cluster (MMC) suggests a densely interconnected module, which often corresponds to core biological functions or signaling pathways critical to disease progression (Islam et al. 2025). Network clustering approaches like MCODE are widely used to detect such functional modules within PPI networks (Szklarczyk et al. 2021).

Biologically, the identified hub targets are well‐known mediators of cell proliferation, survival, inflammation, and drug resistance. For example, EGFR, AKT1, and GSK3B are key components of growth factor‐mediated signaling cascades, while IL6 plays a pivotal role in inflammatory and oncogenic signaling. ESR1/ESR2 are central to hormone‐responsive pathways, and ABCG2 is associated with xenobiotic transport and multidrug resistance. The prominence of these targets within the network supports the hypothesis that the compounds may act via multi‐target mechanisms, aligning with the systems‐level perspective of network pharmacology (Zhang et al. 2019).

### 3.6 Gene Ontology Enrichment and KEGG Pathway Analysis

**FIGURE 7:**
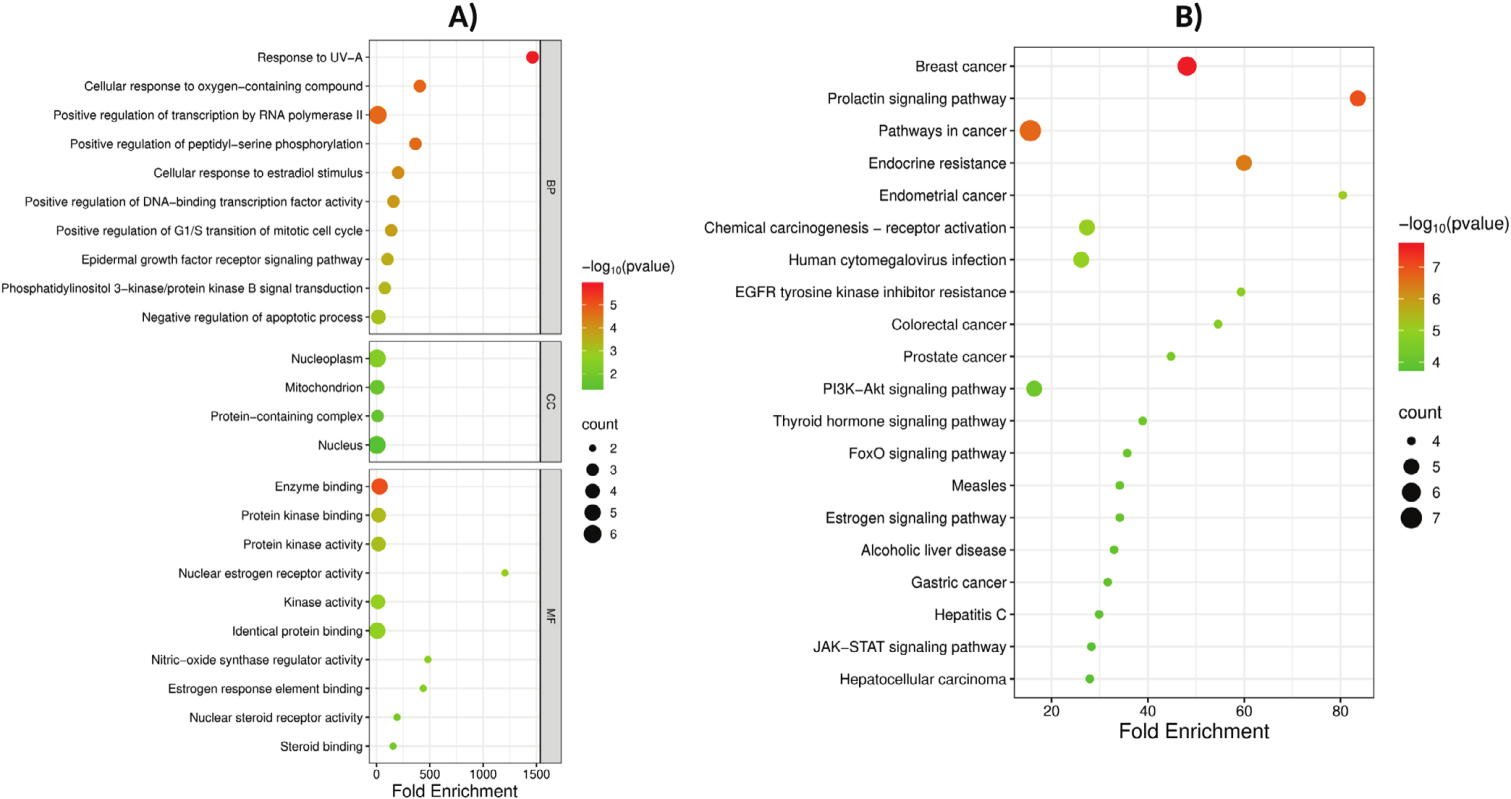
GO and KEGG enrichment analysis of *Euphorbia peplis* : (A) Enriched GO terms related to transcription, oxidative stress, and hormone signaling. (B) Key KEGG pathways involved in cancer are PI3K‐Akt, MAPK, and endocrine resistance.

GO and KEGG analyses are crucial tools for exploring the potential functions of therapeutic targets. GO analysis typically encompasses three domains: molecular function (MF), cellular component (CC), and biological process (BP) (Chen et al. 2015). In this study, the top 10 enriched terms in BPs, CCs, and MFs, as well as the top 20 KEGG signaling pathways, were selected for biological function analysis based on *p* values. In the GO functional annotation analysis of 9 potential targets in *Euphorbia peplis* , a total of 112 terms (*p* < 0.05) were identified, including 46 BPs, 4 CCs, 46 KEGG and 16 MFs. For BPs, the most enriched terms include cellular response to UV‐A, cellular response to oxygen‐containing compounds, cellular response to estradiol stimulus, positive regulation of transcription by RNA, and polymerase II (Figure 7A). In the CC category, the targets were primarily associated with the nucleoplasm, mitochondrion, and protein‐containing complex. These processes are closely associated with cellular adaptation, metabolic regulation, and hormone‐responsive signaling. For MFs, the targets were mainly responsible for enzyme binding, protein kinase binding, and protein kinase activity. The enrichment of oxidative stress‐related and enzyme‐binding terms further suggests that redox balance and PPIs play important roles in the underlying mechanism. Such GO enrichment patterns are commonly observed in studies investigating multitarget compounds acting on complex diseases, where transcriptional regulation and metabolic reprogramming are central events (Tang et al. 2025). In addition, the presence of terms related to nuclear receptor activity supports the involvement of hormone‐mediated signaling pathways. These findings align well with the identification of ESR1 and ESR2 as hub targets, indicating that ligand‐receptor interactions and downstream transcriptional control may represent key modes of action. GO‐based functional annotation is widely used to contextualize target lists and to infer biological relevance at a systems level (Bochalis et al. 2025).

Significantly enriched signaling pathways are associated with the common targets. Key pathways include breast cancer, PI3K‐Akt signaling, prolactin signaling, endocrine resistance, and resistance to EGFR tyrosine kinase inhibitors (Figure 7B). These pathways are closely linked to breast cancer cell proliferation, survival, and therapeutic resistance (Miricescu et al. 2020). The enrichment of MAPK, JAK‐STAT, and FOXO signaling pathways further highlights the critical role of signal transduction cascades that regulate apoptosis, inflammation, and cell cycle progression (Perner et al. 2025; Yue and López 2020; Farooqi et al. 2022). KEGG pathway analysis provides mechanistic insights by mapping targets onto curated signaling pathways, facilitating the biological interpretation of network pharmacology results (Fan et al. 2023).

### 3.7 Compound‐Target‐Pathway Network

**FIGURE 8:**
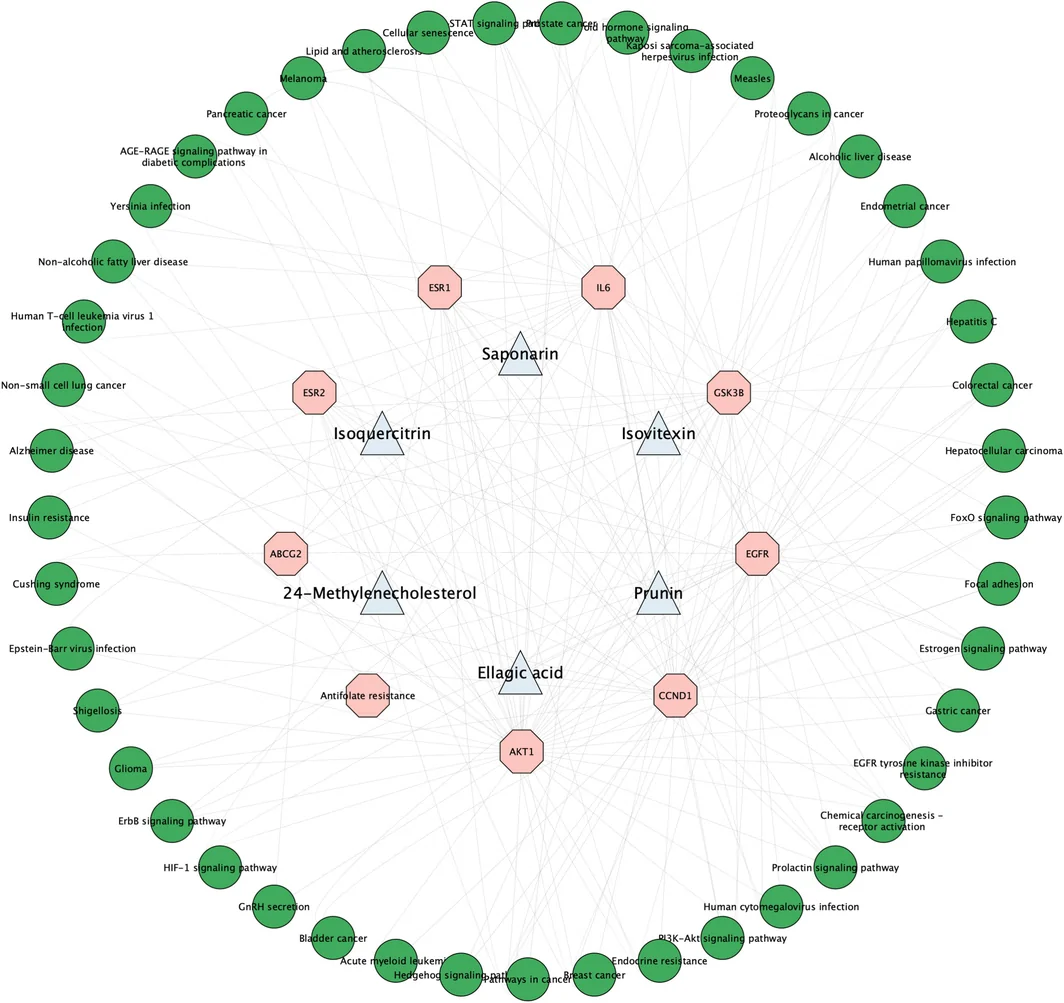
Compound‐target‐pathway interaction network. The network illustrates multi‐component‐multi‐target‐multi‐pathway relationships, highlighting hub targets linking bioactive compounds in *Euphorbia peplis* to cancer‐ and hormone‐related signaling pathways.

The network is organized hierarchically, with bioactive compounds positioned on one side, key protein targets in the central layer, and enriched KEGG pathways on the opposite side (Figure 8). To clarify the molecular basis of the observed biological effects in the SH‐SY5Y neuroblastoma model, a compound‐target‐pathway network was constructed based on the tentatively identified phytochemicals and their predicted targets. Network analysis revealed that isoquercitrin, isovitexin, saponarin, prunin, 24‐methylenecholesterol, and ellagic acid collectively regulate a restricted but functionally critical set of targets. This architecture highlights the characteristic multi‐component‐multi‐target‐multi‐pathway interaction pattern. Central nodes such as AKT1, EGFR, IL6, ESR1/ESR2, CCND1, GSK3B, and ABCG2 show high connectivity, indicating their hub roles in mediating compound‐induced effects. Hub targets typically act as signal integrators and are critical for maintaining network robustness, making them attractive candidates for therapeutic intervention (Hasan et al. 2025).

The pathway layer reveals that many targets converge on cancer‐ and hormone‐related signaling pathways, including PI3K‐Akt signaling, breast cancer, endocrine resistance, EGFR tyrosine kinase inhibitor resistance, and prolactin signaling. The dense interconnections between targets and these pathways suggest strong pathway crosstalk, particularly between growth factor signaling and hormone receptor‐mediated transcriptional regulation. Such crosstalk is a known driver of tumor progression and drug resistance, especially in hormone‐responsive cancers (Yan et al. 2024).

Therefore, the identified target profile is highly consistent with the molecular landscape of SH‐SY5Y cells. Among the compounds, ellagic acid exhibited the highest target connectivity, interacting with AKT1, CCND1, EGFR, ESR1, ESR2, and GSK3B, suggesting a prominent role in modulating kinase‐driven signaling pathways that are critical for SH‐SY5Y cell proliferation and survival. Prunin and 24‐methylenecholesterol were primarily associated with estrogen receptor‐mediated signaling via ESR1 and ESR2, which have been implicated in neuronal differentiation and neuroprotective mechanisms. In contrast, isoquercitrin, isovitexin, and saponarin mainly targeted IL6 and ABCG2, indicating a potential role in the regulation of inflammatory signaling and transporter‐related cellular responses in neuroblastoma cells.

Targets such as ESR1 and ESR2 link multiple compounds to estrogen‐responsive pathways, supporting a potential role in modulating nuclear receptor signaling (Gui et al. 2025). Similarly, AKT1 and GSK3B serve as critical nodes connecting compounds to survival, metabolism, and cell‐cycle regulation pathways, reinforcing their importance in disease‐related signaling cascades. The inclusion of ABCG2 further indicates a possible influence on drug transport and multidrug resistance mechanisms, which are frequently associated with reduced therapeutic efficacy (Wu et al. 2020).

KEGG pathway enrichment analysis revealed significant enrichment of pathways relevant to both cancer biology and neuronal signaling, reflecting the neuroblastoma origin of SH‐SY5Y cells. The most enriched pathways included breast cancer, pathways in cancer, prolactin signaling pathway, and endocrine resistance, all of which converge on core signaling modules such as PI3K‐Akt, EGFR, and cell cycle regulation that are also fundamental to SH‐SY5Y cell biology. Overall, the pathway‐target‐component network shows that the compounds studied influence SH‐SY5Y cells through coordinated regulation of proliferation, survival, and inflammation‐related signaling pathways, rather than through single‐target mechanisms. The consistent involvement of PI3K‐Akt signaling, EGFR‐related pathways, and cell cycle regulation supports a systems‐level mode of action in the SH‐SY5Y neuroblastoma model. These findings offer a solid basis for further molecular docking and molecular dynamics simulations, focusing on key SH‐SY5Y‐relevant hub targets, especially AKT1, EGFR, GSK3B, and CCND1.

### 3.8 Protein‐Ligand Interaction

The predicted binding energy scores and interaction profiles of the most abundant phytochemicals in the extracts of *Euphorbia peplis* against the studied protein targets, along with types and residues involved in different interactions, are shown in Table S1, and detailed interaction analysis is presented below.

AChE: Isoquercitrin exhibited the strongest affinity toward AChE with a docking score of −8.2 kcal/mol, indicating a stable fit within the catalytic gorge. The ligand formed multiple hydrogen bonds with key active‐site residues, including Trp86, Gly120, Gly121, and Glu202, which are well known to line the anionic and catalytic subsites of AChE (He et al. 2025). These polar interactions help anchor the flavonoid core deep in the gorge. Additionally, hydrophobic contacts with aromatic residues such as Trp86 and Tyr337, Phe338, and Tyr341 further stabilized the complex. Such interaction patterns are consistent with reported binding modes of polyphenolic AChE inhibitors and support the favorable docking score observed (Jabir et al. 2018).

BChE: Cycloartenol demonstrated a notable binding affinity toward BChE with a docking score of −10.0 kcal/mol. Unlike polyphenols, this triterpenoid interacted more selectively, forming a hydrogen bond with His438 near the catalytic machinery. The binding was primarily driven by extensive hydrophobic contacts, especially with Trp231, Leu286, and surrounding nonpolar residues, which better accommodate bulky, lipophilic scaffolds in BChE compared to AChE. This hydrophobic dominance is typical of BChE ligands (Miličević and Šinko 2022) and explains the strong docking score despite limited hydrogen bonding.

α‐Glucosidase: Euphol demonstrated high affinity for α‐glucosidase, with a docking score of −9.9 kcal/mol. The ligand formed hydrogen bonds with Glu248 and Arg352, which are residues involved in catalytic activity and substrate stabilization. These interactions likely disrupt normal glycosidic cleavage. Hydrophobic interactions with Leu252, Ile143, and nearby residues helped secure the triterpenoid within the active site. The balance of polar and nonpolar contacts suggests a stable inhibitory pose, similar to previously reported α‐glucosidase inhibitors with comparable scaffolds (Valadbeigi et al. 2025).

Tyrosinase: For tyrosinase, cycloartenol was the best‐scoring compound but with a relatively moderate docking score of −6.2 kcal/mol. Its interaction profile was dominated by hydrophobic contacts with residues such as Val218, Ala221, and His208, reflecting the largely nonpolar nature of the binding region. No strong hydrogen bonds were detected, which likely explains the weaker binding compared to other targets. However, its proximity to histidine residues (H290, H284, H332, and H333) involved in copper coordination suggests potential steric interference with substrate access, a common mechanism observed for non‐chelating tyrosinase inhibitors (Noh et al. 2020).

AKT1: Ellagic acid glucoside and euphol exhibited the strongest interactions with AKT1, both achieving a binding energy of −9.3 kcal/mol, indicating high binding stability within the kinase domain. Ellagic acid glucoside formed multiple hydrogen bonds with Phe161, Gly162, Lys179, and Ala230, residues located near the ATP‐binding pocket, suggesting potential interference with kinase activity. In contrast, euphol formed a hydrogen bond with Gly162 and extensive hydrophobic contacts involving Phe161 and Val164, stabilizing the ligand within the hydrophobic cleft. These interactions are consistent with reported AKT1 inhibitors that target both polar and nonpolar regions of the active site (Manning and Toker 2017).

Cyclin D1: For Cyclin D1, ellagic acid glucoside was the best‐performing ligand with a binding energy of −8.7 kcal/mol. The compound established hydrogen bonds with Glu69 and Lys112, residues involved in the formation of the Cyclin D1‐CDK4/6 complex (Al‐Karmalawy et al. 2025). Additional hydrophobic interactions with Leu68 and Ile84 further anchored the ligand within the regulatory interface. This binding mode suggests a potential to disrupt Cyclin D1‐mediated cell cycle progression, aligning with the known antiproliferative role of Cyclin D1 inhibition (Pellarin et al. 2025).

**FIGURE 9:**
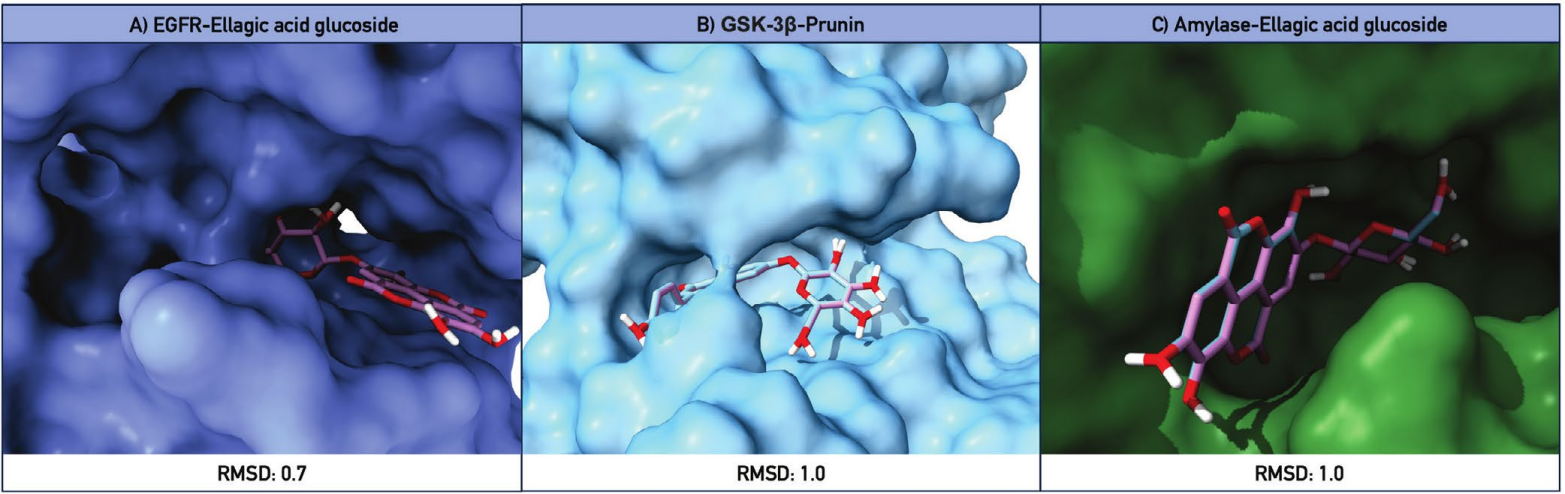
Ligand binding pose: (A) EFGFR‐Ellagic acid glucoside (C1), (B) GSK‐3β‐Prunin (C2), and (C) Amylase‐Ellagic acid glucoside (C3).

EGFR: Ellagic acid glucoside showed the strongest affinity toward EGFR, with a binding energy of −10.5 kcal/mol. The ligand binds deeply within the EGFR pocket with a low RMSD (0.7 Å), indicating a stable pose. The polyphenolic core is well accommodated inside the cavity, while hydroxyl groups orient toward polar residues, supporting anchoring through hydrogen bonding. The glucoside moiety extends toward the pocket entrance, contributing to overall binding stability (Figure 9A). The ligand was stabilized mainly through hydrogen interactions with residues such as Lys721, Glu738, Gln767, and Asp831, residues lining the ATP‐binding pocket of the tyrosine kinase domain. EGFR inhibitors and supports a stable inhibitory pose (Gonçalves et al. 2026).

GSK3β: Prunin demonstrated strong binding to GSK3β, achieving a binding energy of −10.2 kcal/mol and a plausible binding mode. Prunin exhibits a stable binding orientation in the GSK‐3β active site (RMSD 1.0 Å), with its flavonoid core centrally positioned within the pocket. Aromatic regions engage hydrophobic areas, while hydroxyl groups form stabilizing polar interactions, suggesting effective occupation of functionally relevant regions (Figure 9B). The ligand formed hydrogen bonds with key residues—such as Lys85, Glu137, Arg141, Lys183, Gln185—that are critical for ATP coordination (Hua et al. 2023). These polar interactions were complemented by hydrophobic contacts with Ile62 and Val70, reinforcing ligand stability within the active site.

Amylase: Ellagic acid glucoside emerged as the top binder for α‐amylase, achieving a docking score of −10.5 kcal/mol—the strongest among all tested targets. The ligand adopts an extended and stable pose within the amylase active groove (RMSD 1.0 Å). The ellagic acid core penetrates the catalytic pocket, enabling multiple polar contacts, while the glucoside moiety remains solvent‐exposed, potentially contributing to substrate‐blocking activity (Figure 9C). The ligand formed hydrogen bonds with catalytically important residues, including Gln63, Arg195, and Glu233, involved in substrate recognition and proton transfer (Larson et al. 2010). These interactions suggest that the compound effectively occupies the active site and may interfere with polysaccharide binding. Complementary hydrophobic interactions with surrounding residues in the binding cleft further stabilized the complex.

### 3.9 Conformational Stability

**FIGURE 10:**
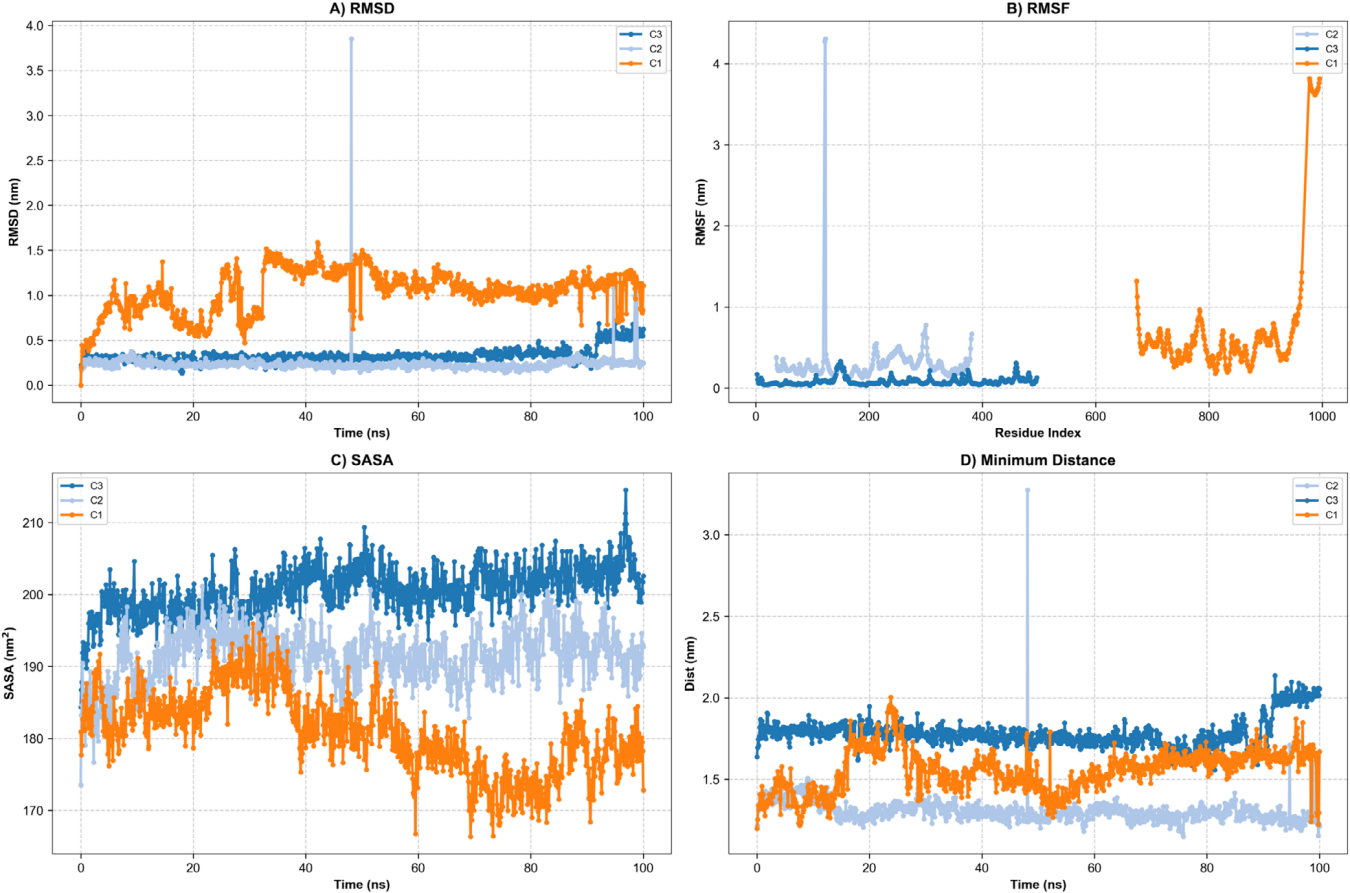
Dynamics of protein‐ligand complexes over 100 ns. (A) RMSD, (B) RMSF, (C) SASA, Distance (A–D). EFGFR‐Ellagic acid glucoside (C1), GSK‐3β‐Prunin (C2), and Amylase‐Ellagic acid glucoside (C3).

RMSD profiles show that all systems remain conformationally stable during the 100 ns simulations, with no signs of large‐ scale structural drift. Amylase‐ellagic acid glucoside has the lowest average RMSD (0.33 Å), indicating a highly rigid and stabilized complex. GSK‐3β‐prunin is even lower on average (0.24 Å) but exhibits a broader maximum deviation, suggesting brief, localized rearrangements rather than ongoing instability. Conversely, EFGFR‐ellagic acid glucoside shows a gradual RMSD increase up to approximately 1.05 Å, consistent with greater overall flexibility (Figure 10A), yet still within a range compatible with a stable protein‐ligand complex (Hollingsworth and Dror 2018).

### 3.10 Residue‐Level Flexibility

RMSF analysis reveals clear differences in local mobility. Amylase‐ellagic acid glucoside displays uniformly low fluctuations (mean 0.08 Å), indicating a rigid backbone and well‐constrained binding environment. GSK‐3β‐prunin exhibits moderate flexibility (mean 0.30 Å), with notable motion at residues 122–123, corresponding to loop regions near the binding site. EFGFR‐ellagic acid glucoside shows the highest residue‐ level fluctuations (mean 0.72 Å), especially at terminal segments (residues 977–995; Figure 10B), suggesting inherent flexibility that does not directly impair ligand binding (Hollingsworth and Dror 2018).

### 3.11 Surface Accessibility and Compactness

SASA values generally remain stable for all complexes, indicating maintained global compactness. Amylase‐ellagic acid glucoside has the highest average SASA (~200.6 nm^2^) with a slight increase over time, consistent with minor surface relaxation (Ali et al. 2014). GSK‐3β‐prunin shows intermediate SASA (~191.4 nm^2^) and a modest rise after 60 ns, reflecting slight solvent exposure changes. EFGFR‐ellagic acid glucoside has the lowest SASA (~180.9 nm^2^) with minimal variation over time (Figure 10C), implying a relatively compact structure despite increased internal flexibility.

### 3.12 Intermolecular Distance

Distance measurements indicate the persistence of ligand binding across all systems. GSK‐3β‐prunin shows the shortest average distance (~1.31 Å) and a 99.9% interaction occupancy, indicating very tight geometric complementarity. EFGFR‐ellagic acid glucoside and amylase‐ellagic acid glucoside maintain slightly longer but still stable distances (~1.55 Å and ~1.78 Å, respectively), each with 100% interaction occupancy (Figure 10D). Maintaining minimum distance through simulation is a reflection of the potential stability of the complexes (Artemenko 2008).

### 3.13 Hydrogen Bond Dynamics

**FIGURE 11:**
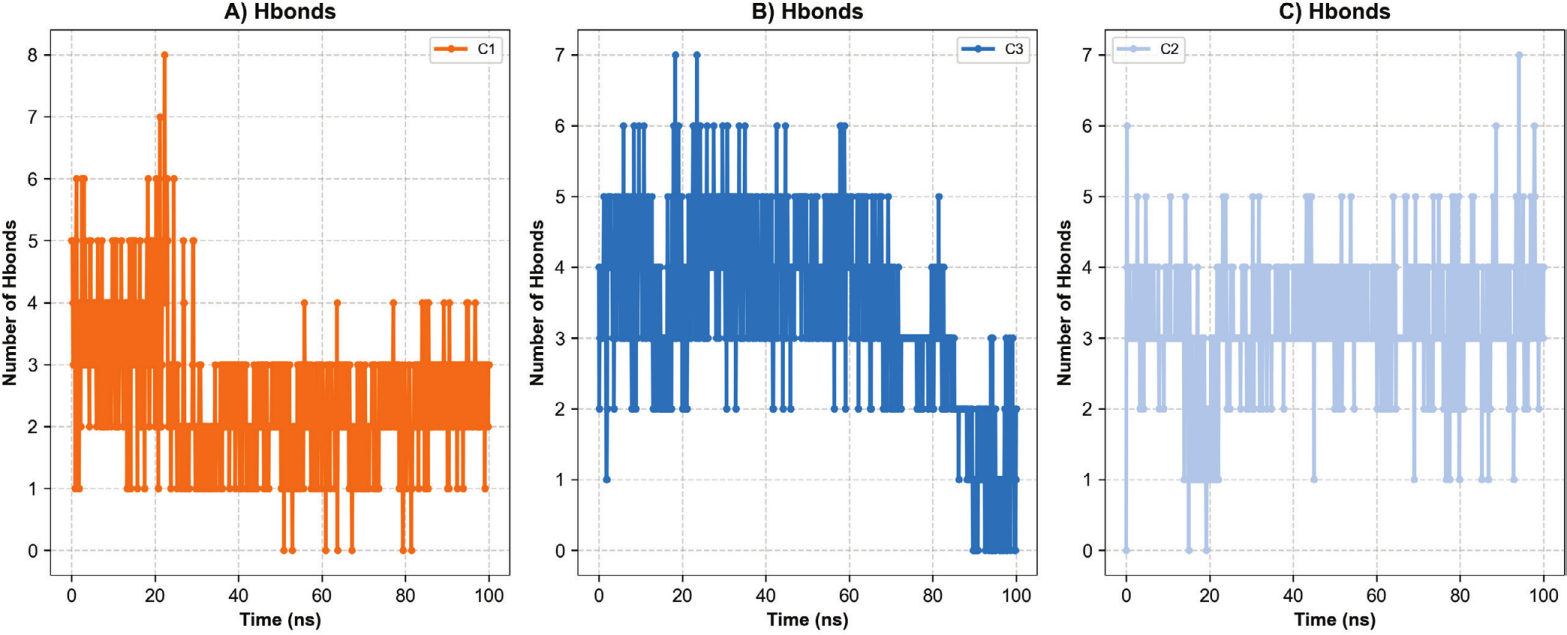
Hydrogen (H‐bond) profiles of EFGFR‐Ellagic acid glucoside (C1), GSK‐3β‐Prunin (C2), and amylase‐Ellagic acid glucoside (C3) over 100 ns.

Hydrogen bond analysis reveals a dynamic interaction network rather than static contacts. Amylase‐ellagic acid glucoside has the highest average hydrogen bond count (4) but shows a significant decreasing trend, implying ongoing rearrangement within the binding interface. GSK‐3β‐prunin shows a moderate average (3) with a slight increasing trend, indicating a gradual optimization of polar contacts over time (Nittinger et al. 2017). EFGFR‐ellagic acid glucoside exhibits the most variability in hydrogen bonding (Figure 11), supporting the idea that its binding stability depends less on persistent hydrogen bonds and more on hydrophobic interactions and steric fit.

Taken together, amylase‐ellagic acid glucoside represents the most rigid and conformationally stable complex, GSK‐3β‐prunin balances strong binding with limited regional flexibility, and EFGFR‐ellagic acid glucoside tolerates higher protein mobility while preserving continuous ligand engagement. The observed instability in hydrogen bond counts across all systems reflects adaptive interaction rearrangements rather than binding weakness, consistent with stable, long‐lived complexes throughout the simulations.

## 4 Conclusion

The present study is the first to report the phytochemical composition and biological activities of *E. peplis* . The extracts were found to be rich in phenolic compounds, and metabolic profiling revealed a diverse array of phytochemicals, including flavonoids, cinnamic acid derivatives, tannins, triterpenoids, and saponins. The extracts exhibited notable antioxidant and enzyme‐inhibitory properties, with the 70% ethanol extract demonstrating the strongest antioxidant activity, while the three organic extracts showed superior enzyme‐inhibitory effects compared to the aqueous extract. The EtOAc extract displayed pronounced cytotoxicity against SHSY5Y cells but also showed toxicity toward normal HEK293 cells. Network pharmacology analysis suggested that *Euphorbia peplis* metabolites may interact with key hub targets, including AKT1, EGFR, GSK3B, ESR1/ESR2, and CCND1. Enrichment analysis highlighted PI3K‐Akt, EGFR, and endocrine resistance pathways, indicating possible modulation of cell survival and proliferation signaling. Molecular docking results showed favorable binding of the metabolites to multiple targets, while MD simulations further indicated stable protein‐ligand complexes with limited structural fluctuations and sustained interactions over time. Overall, the findings indicate that *E. peplis* may serve as a promising source of antioxidants and compounds with potential anticancer and enzyme‐inhibitory effects relevant to various human diseases.

## 5 Limitations

A limitation of the present study is that each solvent extraction was performed only once due to the limited availability of plant material. Consequently, the findings reflect the comparative performance of the tested solvent systems under specific experimental conditions rather than extraction‐to‐extraction variability. Future studies using larger quantities of plant material and independent extraction replicates are warranted to further assess the reproducibility and robustness of the observed phytochemical and biological profiles.

In addition, the phytochemical characterization was based on tentative metabolite annotation using LC–MS/MS fragmentation patterns and comparisons with the literature, without confirmation by authentic reference standards or high‐resolution structural elucidation techniques. Therefore, compound assignments should be interpreted with appropriate caution. The plant material was collected from a single geographic location and over a single sampling period, which may not fully capture the natural chemical variability associated with environmental, seasonal, or geographic factors.

The cytotoxicity assessment was conducted as a preliminary screening at a single concentration (100 μg/mL), and IC_50_ values, dose‐response relationships, and mechanistic investigations were not determined. Furthermore, network pharmacology, molecular docking, and molecular dynamics analyses provide predictive insights into potential mechanisms of action but require experimental validation through target‐based biochemical and cellular studies. Finally, the multivariate analyses were based on metabolite presence–absence patterns rather than quantitative metabolomic data, which may limit the resolution of chemical–bioactivity relationships. Accordingly, future investigations integrating quantitative metabolomics, bioassay‐guided isolation, mechanistic studies, and in vivo validation will be necessary to fully establish the biological significance and therapeutic potential of *Euphorbia peplis* extracts.

## Author Contributions

**Sakina Yagi:** conceptualization, methodology, investigation, writing – original draft, writing – review and editing. **Esraa A. Elhawary:** conceptualization, investigation, methodology, validation, writing – review and editing, writing – original draft, software. **Ilhami Gulcin:** conceptualization, methodology, supervision, writing – original draft, writing – review and editing. **Mehmet Veysi Cetiz:** conceptualization, visualization, writing – original draft, writing – review and editing, methodology. **Omayma A. Eldahshna:** conceptualization, investigation, methodology, writing – review and editing, writing – original draft, supervision. **Ismail Yapıcı:** conceptualization, methodology, validation, writing – review and editing, writing – original draft. **Abdel Nasser B. Singab:** conceptualization, investigation, writing – original draft, writing – review and editing, supervision. **Evren Yildiztugay:** conceptualization, supervision, resources, writing – review and editing. **Maria João Rodrigues:** conceptualization, methodology, investigation, writing – review and editing. **Eliana Fernandes:** conceptualization, investigation, methodology, writing – original draft, software. **Abdullahi Ibrahim Uba:** methodology, visualization, conceptualization, writing – original draft, writing – review and editing. **Luísa Custódio:** conceptualization, investigation, supervision, methodology, writing – review and editing. **Gokhan Zengin:** conceptualization, investigation, methodology, formal analysis, supervision, writing – review and editing, writing – original draft.

## Funding

The authors have nothing to report.

## Conflicts of Interest

The authors declare no conflicts of interest.

## Supporting information


**Table S1:** Docking scores and protein‐ligand interaction of major compounds in *Euphorbia peplis* L. aerial part extracts against selected target proteins.

## Data Availability

The data that support the findings of this study are available on request from the corresponding author.
