# Supplementary material for: Phytochemical Characterization of Euphorbia peplis Extracts and Their Antioxidant, Enzyme Inhibitory, and Cytotoxic Activities: An Integrated Experimental and In Silico Study
Source: Food Sci Nutr. 2026 Aug 26;14(8):e72244. doi: 10.1002/fsn3.72244 (PMC13508148; doi:10.1002/fsn3.72244)
Supplement: Supplementary file 1 [file FSN3-14-e72244-s001.docx]

*Assays for Total Phenolic and Flavonoid Contents*

The total phenolic content was determined by employing the methods given in the literature with some modiﬁcation. Sample solution (0.25 mL) was mixed with diluted Folin–Ciocalteu reagent (1 mL, 1:9, v/v) and shaken vigorously. After 3 min, Na_2_CO_3_ solution (0.75 mL, 1%) was added and the sample absorbance was read at 760 nm after a 2 h incubation at room temperature. The total phenolic content was expressed as milligrams of gallic acid equivalents (mg GAE/g extract)(Uysal et al., 2017).

The total ﬂavonoid content was determined using the AlCl_3_ method. Brieﬂy, sample solution (1 mL) was mixed with the same volume of aluminum trichloride (2%) in methanol. Similarly, a blank was prepared by adding sample solution (1 mL) to methanol (1 mL) without AlCl_3_. The sample and blank absorbances were read at 415 nm after a 10 min incubation at room temperature. The absorbance of the blank was subtracted from that of the sample. Rutin was used as a reference standard and the total ﬂavonoid content was expressed as milligrams of rutin equivalents (mg RE/g extract) (Uysal et al., 2017).

*Determination of Antioxidant and Enzyme Inhibitory Effects*

Antioxidant (DPPH and ABTS radical scavenging, reducing power (CUPRAC and FRAP), phosphomolybdenum and metal chelating (ferrozine method)) and enzyme inhibitory activities (cholinesterase (Elmann’s method), tyrosinase (dopachrome method), α-amylase (iodine/potassium iodide method), α -glucosidase (chromogenic PNPG method) and pancreatic lipase (*p*-nitrophenyl butyrate (p-NPB) method) were determined using the methods previously described by Uysal et al. (Uysal et al., 2017) and Grochowski et al. (Grochowski et al., 2017)

For the DPPH (1,1-diphenyl-2-picrylhydrazyl) radical scavenging assay: Sample solution was added to 4 mL of a 0.004% methanol solution of DPPH. The sample absorbance was read at 517 nm after a 30 min incubation at room temperature in the dark. DPPH radical scavenging activity was expressed as milligrams of trolox equivalents (mg TE/g extract).

For ABTS (2,2′-azino-bis(3-ethylbenzothiazoline) 6-sulfonic acid) radical scavenging assay: Brieﬂy, ABTS+ was produced directly by reacting 7 mM ABTS solution with 2.45 mM potassium persulfate and allowing the mixture to stand for 12–16 in the dark at room temperature. Prior to beginning the assay, ABTS solution was diluted with methanol to an absorbance of 0.700 ± 0.02 at 734 nm. Sample solution was added to ABTS solution (2 mL) and mixed. The sample absorbance was read at 734 nm after a 30 min incubation at room temperature. The ABTS radical scavenging activity was expressed as milligrams of trolox equivalents (mg TE/g extract).

For CUPRAC (cupric ion reducing activity) activity assay: Sample solution was added to premixed reaction mixture containing CuCl_2_ (1 mL, 10 mM), neocuproine (1 mL, 7.5 mM) and NH_4_Ac buffer (1 mL, 1 M, pH 7.0). Similarly, a blank was prepared by adding sample solution (0.5 mL) to premixed reaction mixture (3 mL) without CuCl_2_ . Then, the sample and blank absorbances were read at 450 nm after a 30 min incubation at room temperature. The absorbance of the blank was subtracted from that of the sample. CUPRAC activity was expressed as milligrams of trolox equivalents (mg TE/g extract).

For FRAP (ferric reducing antioxidant power) activity assay: Sample solution was added to premixed FRAP reagent (2 mL) containing acetate buffer (0.3 M, pH 3.6), 2,4,6-tris(2-pyridyl)-S-triazine (TPTZ) (10 mM) in 40 mM HCl and ferric chloride (20 mM) in a ratio of 10:1:1 (v/v/v). Then, the sample absorbance was read at 593 nm after a 30 min incubation at room temperature. FRAP activity was expressed as milligrams of trolox equivalents (mg TE/g extract).

For phosphomolybdenum method: Sample solution was combined with 3 mL of reagent solution (0.6 M sulfuric acid, 28 mM sodium phosphate and 4 mM ammonium molybdate). The sample absorbance was read at 695 nm after a 90 min incubation at 95 °C. The total antioxidant capacity was expressed as millimoles of trolox equivalents (mmol TE/g extract).

For metal chelating activity assay: Brieﬂy, sample solution was added to FeCl_2_ solution (0.05 mL, 2 mM). The reaction was initiated by the addition of 5 mM ferrozine (0.2 mL). Similarly, a blank was prepared by adding sample solution (2 mL) to FeCl_2_ solution (0.05 mL, 2 mM) and water (0.2 mL) without ferrozine. Then, the sample and blank absorbances were read at 562 nm after 10 min incubation at room temperature. The absorbance of the blank was sub-tracted from that of the sample. The metal chelating activity was expressed as milligrams of EDTA (disodium edetate) equivalents (mg EDTAE/g extract).

For Cholinesterase (ChE) inhibitory activity assay: Sample solution (was mixed with DTNB (5,5-dithio-bis(2-nitrobenzoic) acid, Sigma, St. Louis, MO, USA) (125 µL) and AChE (acetylcholines-terase (Electric ell acetylcholinesterase, Type-VI-S, EC 3.1.1.7,Sigma)), or BChE (butyrylcholinesterase (horse serum butyrylcholinesterase, EC 3.1.1.8, Sigma)) solution (25 μL) in Tris–HCl buffer (pH 8.0) in a 96-well microplate and incubated for 15 min at 25 °C. The reaction was then initiated with the addition of acetylthiocholine iodide (ATCI, Sigma) or butyrylthiocholine chloride (BTCl, Sigma) (25 μL). Similarly, a blank was prepared by adding sample solution to all reaction reagents without enzyme (AChE or BChE) solution. The sample and blank absorbances were read at 405 nm after 10 min incubation at 25 °C. The absorbance of the blank was subtracted from that of the sample and the cholinesterase inhibitory activity was expressed as galanthamine equivalents (mgGALAE/g extract).

For Tyrosinase inhibitory activity assay: Sample solution was mixed with tyrosinase solution (40 μL, Sigma) and phosphate buffer (100 μL, pH 6.8) in a 96-well microplate and incubated for 15 min at 25 °C. The reaction was then initiated with the addition of L-DOPA (40 μL, Sigma). Similarly, a blank was prepared by adding sample solution to all reaction reagents without enzyme (tyrosinase) solution. The sample and blank absorbances were read at 492 nm after a 10 min incubation at 25 °C. The absorbance of the blank was subtracted from that of the sample and the tyrosinase inhibitory activity was expressed as kojic acid equivalents (mgKAE/g extract).

For α-amylase inhibitory activity assay: Sample solution was mixed with α-amylase solution (ex-porcine pancreas, EC 3.2.1.1, Sigma) (50 μL) in phosphate buffer (pH 6.9 with 6 mM sodium chloride) in a 96-well microplate and incubated for 10 min at 37 °C. After pre-incubation, the reaction was initiated with the addition of starch solution (50 μL, 0.05%). Similarly, a blank was prepared by adding sample solution to all reaction reagents without enzyme (α-amylase) solution. The reaction mixture was incubated 10 min at 37 °C. The reaction was then stopped with the addition of HCl (25 μL, 1 M). This was followed by addition of the iodine-potassium iodide solution (100 μL). The sample and blank absorbances were read at 630 nm. The absorbance of the blank was subtracted from that of the sample and the α-amylase inhibitory activity was expressed as acarbose equivalents (mmol ACE/g extract).

For α-glucosidase inhibitory activity assay: Sample solution was mixed with glutathione (50 µL), α-glucosidase solution (from Saccharomyces cerevisiae, EC 3.2.1.20, Sigma) (50 µL) in phosphate buffer (pH 6.8) and PNPG (4-N-trophenyl-α-D-glucopyranoside, Sigma) (50 µL) in a 96-well microplate and incubated for 15 min at 37 °C. Similarly, a blank was prepared by adding sample solution to all reaction reagents without enzyme (α-glucosidase) solution. The reaction was then stopped with the addition of sodium carbonate (50 µL, 0.2 M). The sample and blank absorbances were read at 400 nm. The absorbance of the blank was subtracted from that of the sample and the α-glucosidase inhibitory activity was expressed as acarbose equivalents (mmol ACE/g extract).

**References**

Grochowski, D. M., Uysal, S., Aktumsek, A., Granica, S., Zengin, G., Ceylan, R., . . . Tomczyk, M. (2017). In vitro enzyme inhibitory properties, antioxidant activities, and phytochemical profile of Potentilla thuringiaca. *Phytochemistry Letters, 20*, 365-372.

Uysal, S., Zengin, G., Locatelli, M., Bahadori, M. B., Mocan, A., Bellagamba, G., . . . Aktumsek, A. (2017). Cytotoxic and enzyme inhibitory potential of two Potentilla species (P. speciosa L. and P. reptans Willd.) and their chemical composition. *Frontiers in pharmacology, 8*, 290.

**Table S1**. Docking scores and protein-ligand interaction of major compounds in *Euphorbia peplis L*. aerial part extracts against selected target proteins.

| **Compound** | **Target** | **PDB ID** | **Binding Energy**  **(kcal/mol)** | **RMSD (Å)** | **Type of Interaction and Interacting Residues** |
| --- | --- | --- | --- | --- | --- |
| 24-methylenecholesterol | AChE | 7E3H | -4.9 | 1.1 | Hbond: ALA:127 Hydrophobic: TRP:86;TRP:86;LEU:130;TRP:286;VAL:294;PHE:297;TYR:337;TYR:337;  TYR:337;PHE:338;PHE:338;TYR:341;TYR:341;TYR:341 |
| Cycloartenol | AChE | 7E3H | -4.1 | 1.0 | Hbond: TYR:133 Hydrophobic: TYR:72;TYR:72;ASP:74;TRP:86;TRP:86;TRP:86;TYR:124;TRP:286;  TYR:337;TYR:337;TYR:337;PHE:338;TYR:341;TYR:341;ILE:451 |
| Ellagic acid glucoside | AChE | 7E3H | -7.7 | 1.0 | Hbond: GLY:120;TYR:124;SER:125;TYR:133;TYR:133;PHE:295 Pi-stacking: TRP:86;TRP:86;TRP:86;TRP:86;TRP:86 Salt bridge: HIS:447 |
| Isoquercitrin | AChE | 7E3H | -8.2 | 0.5 | Hbond: TRP:86;GLY:120;GLY:121;TYR:124;TYR:124;SER:125;GLY:126;  TYR:133;GLU:202;PHE:295;HIS:447 Hydrophobic: TRP:86;TYR:337;PHE:338;TYR:341 |
| Prunin | AChE | 7E3H | -6.2 | 0.9 | Hbond: TYR:72;ASP:74;ASP:74;ASN:87;GLY:120;TYR:124;GLY:126;TYR:133;PHE:338 Hydrophobic: TRP:86;TYR:337;TYR:337;TYR:341 Pi-stacking: PHE:338 |
| 24-methylenecholesterol | Amylase | 2qv4 | -9.0 | 1.0 | Hydrophobic: TRP:58;TRP:59;TRP:59;TRP:59;TRP:59;TRP:59;TRP:59;TYR:62;TYR:62;  LEU:162;LEU:165;ALA:198 |
| 24-methylenecycloartanol | Amylase | 2qv4 | -9.9 | 0.8 | Hydrophobic: TRP:59;TRP:59;TYR:62;LEU:162;LEU:165;ILE:235 |
| Cycloartenol | Amylase | 2qv4 | -10.3 | 0.0 | Hydrophobic: TRP:59;TRP:59;TRP:59;TYR:62;LEU:162;LEU:165;  LEU:165;LYS:200;ILE:235;ILE:235 |
| Ellagic acid glucoside | Amylase | 2qv4 | -10.5 | 1.0 | Hbond: GLN:63;GLN:63;ARG:195;GLU:233 Hydrophobic: TRP:59 Pi-stacking: TRP:59;TRP:59;TRP:59 Salt bridge: HIS:305 |
| Euphol | Amylase | 2qv4 | -9.6 | 0.4 | Hbond: ARG:195;GLU:233 Hydrophobic: TRP:59;TRP:59;TRP:59;TRP:59;TYR:62;TYR:62;  TYR:62;LEU:162;THR:163;LEU:165;ALA:198 |
| Friedelin | Amylase | 2qv4 | -10.2 | 0.0 | Hbond: LYS:200;HIS:201 Hydrophobic: TRP:59;TRP:59;TRP:59;TYR:62;LEU:162;ILE:235 |
| Gypsogenin | Amylase | 2qv4 | -9.2 | 0.8 | Hbond: THR:163 Hydrophobic: TYR:62;TYR:62;TYR:62;LEU:162;LEU:162;ILE:235 |
| Isoquercitrin | Amylase | 2qv4 | -8.1 | 0.6 | Hbond: TRP:59;GLN:63;ARG:195;ASP:197;HIS:299;HIS:305;HIS:305 Hydrophobic: THR:163;LEU:165 |
| Isovitexin | Amylase | 2qv4 | -8.9 | 1.0 | Hbond: GLN:63;ARG:195 Hydrophobic: TYR:62;TYR:62;LEU:165 |
| Prunin | Amylase | 2qv4 | -9.5 | 0.6 | Hbond: ALA:198;GLU:233 Hydrophobic: TRP:58;TRP:59;TRP:59;LEU:165 |
| Saponarin | Amylase | 2qv4 | -8.4 | 11.6 | Hbond: GLN:63;GLY:164;ARG:195;GLU:233;HIS:305 Hydrophobic: TYR:62;TYR:62;LEU:165 |
| 24-methylenecholesterol | BChE | 6EQP | -9.6 | 1.0 | Hbond: TRP:82;TRP:430 Hydrophobic: TRP:231;TRP:231;LEU:286;VAL:288;PHE:329;PHE:329;TYR:332;  PHE:398;TRP:430 |
| 24-methylenecycloartanol | BChE | 6EQP | -9.5 | 0.1 | Hydrophobic: TRP:231;TRP:231;LEU:286;VAL:288;  PHE:329;TYR:332;PHE:398 |
| Cycloartenol | BChE | 6EQP | -10.0 | 1.1 | Hbond: HIS:438 Hydrophobic: GLN:119;TRP:231;TRP:231;LEU:286;VAL:288;PHE:329;PHE:329;  TYR:332;PHE:398;TRP:430;HIS:438 |
| Ellagic acid glucoside | BChE | 6EQP | -9.9 | 1.0 | Hbond: ASP:70;TRP:82;SER:198;TYR:332;TRP:430;HIS:438 Pi-stacking: PHE:329 Salt bridge: HIS:438 |
| Euphol | BChE | 6EQP | -8.3 | 1.0 | Hydrophobic: TRP:82;TRP:82;GLN:119;TRP:231;TRP:231;LEU:286;VAL:288;  ALA:328;PHE:329;TYR:332;PHE:398 |
| Friedelin | BChE | 6EQP | -6.6 | 0.0 | Hbond: TRP:82;TRP:430 Hydrophobic: TRP:231;TRP:231;LEU:286;VAL:288;PHE:329;  PHE:329;TYR:332;TYR:440 |
| Gypsogenin | BChE | 6EQP | -4.8 | 0.9 | Hydrophobic: TRP:231;LEU:286;VAL:288;ALA:328;PHE:329;PHE:329;  PHE:398;TRP:430;TYR:440 |
| Isoquercitrin | BChE | 6EQP | -9.6 | 0.5 | Hbond: TRP:82;GLY:115;GLY:115;GLY:115;GLY:115;THR:120;THR:120;TYR:128 Hydrophobic: PHE:329;TYR:332;TRP:430 |
| Isovitexin | BChE | 6EQP | -1.8 | 0.3 | Hbond: ASP:70;ASP:70;TRP:82;SER:287;ASN:289;ALA:328;TRP:430;HIS:438 Hydrophobic: GLN:119;VAL:288;PHE:329 |
| Prunin | BChE | 6EQP | -8.9 | 1.0 | Hbond: ASP:70;ASP:70;THR:120;THR:120;GLU:197;LEU:286;TYR:332 Hydrophobic: ASP:70;VAL:288;PHE:329 Pi-stacking: TRP:82 |
| Saponarin | BChE | 6EQP | -4.5 | 0.3 | Hbond: ASP:70;ASP:70;TRP:82;GLY:115;GLY:115;THR:120;TYR:128;  GLU:197;ALA:328;TRP:430 Hydrophobic: TRP:231;LEU:286;VAL:288;PHE:329 Pi-stacking: PHE:329 |
| 24-methylenecholesterol | Glucosidase | 7KBJ | -8.6 | 1.0 | Hydrophobic: MET:289;TYR:291;ILE:327;PHE:330;PHE:330;TRP:331;  LEU:344;TYR:348;PHE:350 |
| 24-methylenecycloartanol | Glucosidase | 7KBJ | -8.6 | 1.6 | Hbond: LYS:283 Hydrophobic: LEU:252;LEU:252;ILE:257;TYR:291;ILE:327;TRP:331;TYR:348;PHE:350 |
| Cycloartenol | Glucosidase | 7KBJ | -8.7 | 0.5 | Hbond: LYS:283 Hydrophobic: LEU:252;LEU:252;ILE:257;TYR:291;TRP:331;TYR:348 |
| Ellagic acid glucoside | Glucosidase | 7KBJ | -9.5 | 0.6 | Hbond: GLU:248;LYS:283;TYR:348;PHE:350 Hydrophobic: LEU:252 Salt bridge: ARG:352 Pi-cation: ARG:352 |
| Euphol | Glucosidase | 7KBJ | -9.9 | 1.5 | Hbond: GLU:248;ARG:352 Hydrophobic: LEU:252;ILE:257;PHE:330;PHE:330;TYR:348;PHE:350 |
| Friedelin | Glucosidase | 7KBJ | -4.4 | 10.1 | Hbond: ALA:345 Hydrophobic: LEU:252;LEU:252;ILE:257;TYR:291;TYR:291 |
| Gypsogenin | Glucosidase | 7KBJ | -7.6 | 0.7 | Hbond: GLU:248;LYS:283;GLU:287;TYR:291;TYR:291;ARG:334;ARG:352 Hydrophobic: LEU:252;TYR:291;PHE:350 |
| Isoquercitrin | Glucosidase | 7KBJ | -8.1 | 2.7 | Hbond: GLN:247;ASP:251;LEU:252;GLY:253;GLY:254;GLY:264;TYR:291;TYR:291;TYR:291 Hydrophobic: PHE:350 Pi-cation: ARG:352 |
| Isovitexin | Glucosidase | 7KBJ | -9.5 | 0.3 | Hbond: GLN:247;ASP:251;LEU:252;GLY:253;GLY:254;LYS:283;ALA:345;  TYR:348;TYR:348;ARG:352;ARG:352;ARG:352 Hydrophobic: TYR:291;LEU:344 |
| Prunin | Glucosidase | 7KBJ | -8.9 | 1.0 | Hbond: TYR:246;ASP:251;GLY:253;GLY:266;LYS:283;SER:290;SER:290;  LEU:344;ALA:345;ARG:352;ARG:352 Salt bridge: ARG:352 Pi-cation: ARG:352 |
| Saponarin | Glucosidase | 7KBJ | -5.2 | 0.0 | Hbond: GLN:247;GLY:253;GLY:264;LYS:283;GLU:287;GLU:287;  GLU:287;SER:290;ALA:345;PHE:350;ARG:352 Hydrophobic: LEU:344;PHE:350 Salt bridge: ARG:352 |
| 24-methylenecholesterol | Tyrosinase | 6QXD | -5.9 | 0.5 | Hydrophobic: PHE:197;VAL:218;VAL:218;ALA:221 |
| 24-methylenecycloartanol | Tyrosinase | 6QXD | -5.8 | 1.3 | Hydrophobic: HIS:208;ARG:209;VAL:218;VAL:218;VAL:218 |
| Cycloartenol | Tyrosinase | 6QXD | -6.2 | 0.8 | Hydrophobic: ASN:205;HIS:208;VAL:218;VAL:218;ALA:221 |
| Ellagic acid glucoside | Tyrosinase | 6QXD | -5.4 | 0.7 | Hbond: ASN:205;GLY:216;VAL:217 Pi-cation: ARG:209 |
| Euphol | Tyrosinase | 6QXD | -6.0 | 3.6 | Hbond: ASN:205 Hydrophobic: ASN:205;HIS:208;ARG:209;VAL:217;VAL:218;VAL:218 |
| Friedelin | Tyrosinase | 6QXD | -4.1 | 0.1 | Hbond: GLU:195 Hydrophobic: HIS:60;PHE:197;VAL:217;VAL:218 |
| Gypsogenin | Tyrosinase | 6QXD | -1.8 | 0.5 | Hydrophobic: HIS:208;VAL:218;VAL:218 Salt bridge: ARG:209 |
| Isoquercitrin | Tyrosinase | 6QXD | -5.0 | 0.6 | Hbond: ASN:205;ASN:205;ASN:205;ARG:209;ARG:209;ARG:209;MET:215 Hydrophobic: VAL:217;VAL:218 |
| Prunin | Tyrosinase | 6QXD | -5.1 | 0.7 | Hbond: ARG:209 Hydrophobic: VAL:218;ALA:221 Pi-stacking: HIS:208 |
| 24-methylenecholesterol | AKT1 | 4GV1 | -8.2 | 0.4 | Hydrophobic: LEU:156;PHE:161;VAL:164;VAL:164;ALA:177;TYR:229;THR:291;PHE:438 |
| 24-methylenecycloartanol | AKT1 | 4GV1 | -8.6 | 1.0 | Hbond: THR:160 Hydrophobic: PHE:161;VAL:164;ALA:177;TYR:229;ALA:230;THR:291 |
| Cycloartenol | AKT1 | 4GV1 | -7.9 | 1.0 | Hydrophobic: PHE:161;PHE:161;ASP:292;LEU:295 |
| Ellagic acid glucoside | AKT1 | 4GV1 | -9.3 | 0.2 | Hbond: PHE:161;GLY:162;GLY:162;LYS:179;ALA:230 Hydrophobic: LEU:156;VAL:164;VAL:164;PHE:438 Salt bridge: LYS:179;LYS:179 |
| Euphol | AKT1 | 4GV1 | -9.3 | 1.0 | Hbond: GLY:162 Hydrophobic: PHE:161;VAL:164;GLU:234;PHE:236;PHE:237;GLU:278;GLU:278;PHE:438;ASP:439;PHE:442 |
| Friedelin | AKT1 | 4GV1 | -9.0 | 0.1 | Hydrophobic: PHE:161;VAL:164;LEU:295;LEU:295 |
| Gypsogenin | AKT1 | 4GV1 | -8.6 | 1.1 | Hbond: THR:312 Hydrophobic: PHE:161;VAL:164;LYS:179;THR:312 Salt bridge: LYS:276 |
| Isoquercitrin | AKT1 | 4GV1 | -8.6 | 0.6 | Hbond: GLY:157;PHE:161;GLY:162;LYS:179;GLU:198;LYS:276;ASP:292;ASP:292;GLY:294;GLY:311 Hydrophobic: PHE:161;LEU:181 |
| Isovitexin | AKT1 | 4GV1 | -8.1 | 0.5 | Hbond: GLY:159;PHE:161;GLY:162;GLY:162;ASP:439 Hydrophobic: PHE:236;ASP:439;PHE:442 |
| Prunin | AKT1 | 4GV1 | -9.0 | 1.0 | Hbond: LYS:158;ALA:230;ASP:274;LYS:276;ASN:279 Hydrophobic: LEU:156;LEU:156;VAL:164;VAL:164;PHE:438 Salt bridge: LYS:179 Pi-cation: LYS:179 |
| Saponarin | AKT1 | 4GV1 | -9.4 | 1.0 | Hbond: PHE:161;GLY:162;GLY:162;LYS:179;ASP:292;ASP:292;ASP:439 Hydrophobic: PHE:236;ASP:439;PHE:442 |
| 24-methylenecholesterol | Cyclin D1 | 2w99 | -7.5 | 0.8 | Hydrophobic: LEU:65;GLU:69;LYS:72;GLU:75;PHE:78;GLN:183 |
| 24-methylenecycloartanol | Cyclin D1 | 2w99 | -7.3 | 0.7 | Hbond: GLN:176 Hydrophobic: LEU:65;GLU:75;PHE:78 |
| Cycloartenol | Cyclin D1 | 2w99 | -7.4 | 10.2 | Hbond: GLN:176 Hydrophobic: LEU:65;GLU:69;PRO:79;ALA:187 |
| Ellagic acid glucoside | Cyclin D1 | 2w99 | -7.8 | 1.0 | Hbond: CYS:73;GLN:176;GLN:176;ARG:179;ARG:179;LYS:180;THR:184 Hydrophobic: LEU:65;GLU:75 Salt bridge: LYS:180 |
| Euphol | Cyclin D1 | 2w99 | -8.0 | 0.9 | Hydrophobic: GLU:69;GLU:75;GLU:75;PRO:79;LYS:180;ALA:187 |
| Friedelin | Cyclin D1 | 2w99 | -8.4 | 0.0 | Hydrophobic: GLU:69;LYS:72;GLU:75;PHE:78;GLN:183 |
| Gypsogenin | Cyclin D1 | 2w99 | -7.4 | 1.1 | Hbond: PHE:78 Hydrophobic: LEU:65;GLU:69;GLU:75;LYS:180 Salt bridge: LYS:180 |
| Isoquercitrin | Cyclin D1 | 2w99 | -7.2 | 0.6 | Hbond: CYS:73;GLU:74;GLU:75;LYS:180;GLN:183 Hydrophobic: LYS:180 |
| Isovitexin | Cyclin D1 | 2w99 | -7.1 | 0.1 | Hbond: CYS:68;LYS:72;HIS:158;THR:184 Hydrophobic: GLU:69;LYS:72;LYS:72 |
| Prunin | Cyclin D1 | 2w99 | -8.2 | 0.8 | Hbond: CYS:73;GLU:74;GLU:75;PHE:78;GLN:176 Hydrophobic: LYS:180;GLN:183 |
| Saponarin | Cyclin D1 | 2w99 | -8.2 | 0.7 | Hbond: CYS:73;GLU:74;LYS:180;GLN:183;THR:184;THR:191;GLN:261;GLN:261 Hydrophobic: LEU:65;LEU:65;LEU:188 |
| 24-methylenecholesterol | EGFR | 1m17 | -9.3 | 1.0 | Hydrophobic: LEU:694;LEU:694;LEU:694;VAL:702;ALA:719;LYS:721;LYS:721;LEU:764;LEU:820 |
| 24-methylenecycloartanol | EGFR | 1m17 | -8.7 | 0.3 | Hydrophobic: LEU:694;LEU:694;LEU:694;PHE:699;PHE:699;VAL:702;VAL:702;LEU:768;LEU:820 |
| Cycloartenol | EGFR | 1m17 | -8.8 | 1.0 | Hydrophobic: LEU:694;LEU:694;PHE:699;PHE:699;PHE:699;VAL:702;LEU:820 |
| Ellagic acid glucoside | EGFR | 1m17 | -10.5 | 0.7 | Hbond: LEU:694;LYS:721;LYS:721;GLU:738;THR:766;GLN:767;MET:769;GLY:772;THR:830;THR:830;ASP:831;ASP:831 Hydrophobic: LEU:694;LEU:694;VAL:702 |
| Euphol | EGFR | 1m17 | -8.6 | 0.7 | Hydrophobic: LEU:694;LEU:694;VAL:702;ALA:719;LYS:721;LYS:721;LEU:764;THR:766;LEU:820;LEU:820;THR:830 |
| Friedelin | EGFR | 1m17 | -9.3 | 0.0 | Hydrophobic: LEU:694;PHE:699;PHE:699;VAL:702;VAL:702;ARG:817;LEU:820 |
| Gypsogenin | EGFR | 1m17 | -8.8 | 0.7 | Hbond: ALA:719;GLU:738 Hydrophobic: VAL:702;ALA:719;LYS:721;LYS:721;THR:766;LEU:820;LEU:820 |
| Isoquercitrin | EGFR | 1m17 | -9.7 | 0.6 | Hbond: ALA:719;LYS:721;LYS:721;GLU:738;THR:766;MET:769;GLY:772;ASP:831;ASP:831;ASP:831 Hydrophobic: VAL:702;ALA:719;THR:766 |
| Isovitexin | EGFR | 1m17 | -9.4 | 0.5 | Hbond: LYS:721;GLU:738;GLU:738;LEU:764;THR:766;THR:830;ASP:831;ASP:831;ASP:831 Hydrophobic: LEU:694;ALA:719;LEU:820 |
| Prunin | EGFR | 1m17 | -9.2 | 1.0 | Hbond: LYS:721;LYS:721;THR:766 Hydrophobic: PHE:699;VAL:702;ALA:719;LYS:721;LYS:721 Salt bridge: LYS:721 |
| Saponarin | EGFR | 1m17 | -9.4 | 0.9 | Hbond: ALA:719;LYS:721;GLU:738;THR:766;THR:766;MET:769;THR:830;ASP:831;ASP:831;ASP:831 Hydrophobic: LEU:694;ALA:719;LEU:768 |
| 24-methylenecholesterol | GSK3B | 8QJI | -8.7 | 0.9 | Hbond: GLN:185 Hydrophobic: VAL:70;VAL:70;LYS:85;VAL:110;LEU:132;LEU:132;TYR:140;GLN:185;GLN:185;LEU:188;LEU:188 Salt bridge: ARG:220;ARG:223 Pi-cation: ARG:223 |
| 24-methylenecycloartanol | GSK3B | 8QJI | -9.0 | 4.9 | Hydrophobic: ILE:62;ILE:62;VAL:70;VAL:70;ALA:83;LYS:85;LYS:85;VAL:110;LEU:132;THR:138;GLN:185;LEU:188 Salt bridge: ARG:220;ARG:223 Pi-cation: ARG:223 |
| Cycloartenol | GSK3B | 8QJI | -9.1 | 0.4 | Hydrophobic: ILE:62;PHE:67;PHE:67;VAL:70;VAL:110;GLN:185;LEU:188;LEU:188;ASP:200 Salt bridge: ARG:220;ARG:223 Pi-cation: ARG:223 |
| Ellagic acid glucoside | GSK3B | 8QJI | -10.0 | 0.6 | Hbond: ILE:62;VAL:135;PRO:136;GLU:137;ARG:141;ARG:141 Hydrophobic: ILE:62;VAL:70;LYS:85;LEU:188 Salt bridge: ARG:220;ARG:223;ARG:141 Pi-cation: ARG:223 |
| Euphol | GSK3B | 8QJI | -8.6 | 0.9 | Hbond: THR:275;GLN:280 Hydrophobic: ILE:256;PHE:257;LEU:273;PRO:300;THR:302;PHE:305;LEU:321;TYR:323 Salt bridge: ARG:220;ARG:223 Pi-cation: ARG:223 |
| Friedelin | GSK3B | 8QJI | -8.8 | 36.5 | Hydrophobic: PHE:229;LEU:266;ILE:270;ILE:281;PRO:286;PHE:291 Salt bridge: ARG:220;ARG:223 Pi-cation: ARG:223 |
| Gypsogenin | GSK3B | 8QJI | -8.2 | 0.8 | Hydrophobic: ILE:62;ILE:62;TYR:134;GLN:185;LEU:188 Salt bridge: ARG:220;ARG:223 Pi-cation: ARG:223 |
| Isoquercitrin | GSK3B | 8QJI | -8.5 | 0.7 | Hbond: LYS:85;ASP:133;VAL:135;THR:138;ARG:141;ARG:141;LYS:183;ASN:186;ASN:186;ASP:200;ASP:200 Hydrophobic: VAL:70;ALA:83;LEU:188 Salt bridge: ARG:220;ARG:223 Pi-cation: ARG:223 |
| Isovitexin | GSK3B | 8QJI | -8.6 | 0.9 | Hbond: SER:66;SER:66;PHE:67;LYS:85;VAL:135;LYS:183;ASN:186;ASN:186;ASP:200 Hydrophobic: ILE:62;VAL:70;LEU:188 Salt bridge: ARG:220;ARG:223 Pi-cation: ARG:223 |
| Prunin | GSK3B | 8QJI | -10.2 | 1.0 | Hbond: LYS:85;VAL:135;GLU:137;ARG:141;LYS:183;GLN:185 Hydrophobic: ILE:62;ILE:62;VAL:70;VAL:70;TYR:134;TYR:134;THR:138 Salt bridge: ARG:220;ARG:223 Pi-cation: ARG:223 |
| Saponarin | GSK3B | 8QJI | -9.1 | 0.2 | Hbond: ILE:62;TYR:134;VAL:135;VAL:135;GLU:137;ARG:141;ARG:141;CYS:199 Hydrophobic: VAL:70 Pi-stacking: PHE:67 Salt bridge: ARG:220;ARG:223 Pi-cation: ARG:223;LYS:183 |
